# The Amino Terminal Domain and Modulation of Connexin36 Gap Junction Channels by Intracellular Magnesium Ions

**DOI:** 10.3389/fphys.2022.839223

**Published:** 2022-02-21

**Authors:** Tadas Kraujalis, Lukas Gudaitis, Lina Kraujaliene, Mindaugas Snipas, Nicolás Palacios-Prado, Vytas K. Verselis

**Affiliations:** ^1^Institute of Cardiology, Lithuanian University of Health Sciences, Kaunas, Lithuania; ^2^Department of Applied Informatics, Kaunas University of Technology, Kaunas, Lithuania; ^3^Department of Mathematical Modelling, Kaunas University of Technology, Kaunas, Lithuania; ^4^Department of Physiology, Pontificia Universidad Católica de Chile, Santiago, Chile; ^5^Centro Interdisciplinario de Neurociencias de Valparaíso, Universidad de Valparaiso, Valparaíso, Chile; ^6^Dominick P. Purpura Department of Neuroscience, Albert Einstein College of Medicine, New York, NY, United States

**Keywords:** gap junction, connexin, intracellular Mg^2+^, ion permeability, electrophysiology

## Abstract

Electrical synapses between neurons in the mammalian CNS are predominantly formed of the connexin36 (Cx36) gap junction (GJ) channel protein. Unique among GJs formed of a number of other members of the Cx gene family, Cx36 GJs possess a high sensitivity to intracellular Mg^2+^ that can robustly act to modulate the strength of electrical synaptic transmission. Although a putative Mg^2+^ binding site was previously identified to reside in the aqueous pore in the first extracellular (E1) loop domain, the involvement of the N-terminal (NT) domain in the atypical response of Cx36 GJs to pH was shown to depend on intracellular levels of Mg^2+^. In this study, we examined the impact of amino acid substitutions in the NT domain on Mg^2+^ modulation of Cx36 GJs, focusing on positions predicted to line the pore funnel, which constitutes the cytoplasmic entrance of the channel pore. We find that charge substitutions at the 8th, 13th, and 18th positions had pronounced effects on Mg^2+^ sensitivity, particularly at position 13 at which an A13K substitution completely abolished sensitivity to Mg^2+^. To assess potential mechanisms of Mg^2+^ action, we constructed and tested a series of mathematical models that took into account gating of the component hemichannels in a Cx36 GJ channel as well as Mg^2+^ binding to each hemichannel in open and/or closed states. Simultaneous model fitting of measurements of junctional conductance, g_j_, and transjunctional Mg^2+^ fluxes using a fluorescent Mg^2+^ indicator suggested that the most viable mechanism for Cx36 regulation by Mg^2+^ entails the binding of Mg^2+^ to and subsequent stabilization of the closed state in each hemichannel. Reduced permeability to Mg^2+^ was also evident, particularly for the A13K substitution, but homology modeling of all charge-substituted NT variants showed only a moderate correlation between a reduction in the negative electrostatic potential and a reduction in the permeability to Mg^2+^ ions. Given the reported role of the E1 domain in Mg^2+^ binding together with the impact of NT substitutions on gating and the apparent state-dependence of Mg^2+^ binding, this study suggests that the NT domain can be an integral part of Mg^2+^ modulation of Cx36 GJs likely through the coupling of conformational changes between NT and E1 domains.

## Introduction

Gap junction (GJ) channels, formed of connexin (Cx) protein subunits, serve as intercellular communication pathways that enable the efficient propagation of electrical signals and the flux of small ions, amino acids, metabolites and peptides between cells (Niessen et al., 2000; Neijssen et al., 2005; Bedner et al., 2006). There are 20 Cxs expressed in rodents and 21 in humans and the Cx36 isoform is the main Cx expressed in neurons thereby constituting the bulk of the electrical synapses in the adult brain, as well as in retinal circuits (Connors and Long, 2004). Cx36 also has been identified in GJs between islet beta cells in rodent (Orci et al., 1973; Meda et al., 1979; Int Veld et al., 1986) and human pancreas (Serre-Beinier et al., 2008), where it plays a key role in regulating glucose homeostasis (Pérez-Armendariz, 2013).

Of particular relevance to electrical synapses is the extent to which the strength of electrical coupling can be modulated, which can substantially impact neuronal synchronization and the coordination of activity within neuronal networks (Bennett and Zukin, 2004; Bissiere et al., 2011). Forms of modulation include voltage dependence, expressed as a sensitivity to the transjunctional voltage (V_j_), the voltage difference between cells (Harris et al., 1981; Bukauskas and Verselis, 2004) and chemical factors, most notably intracellular H^+^ (Trexler et al., 1999; Palacios-Prado et al., 2010) and intracellular divalent cations such as Ca^2+^ and Mg^2+^ (Noma and Tsuboi, 1987; Peracchia, 2004; Matsuda et al., 2010; Palacios-Prado et al., 2013). These forms of modulation are common among GJ channels, but sensitivities can differ substantially among the various Cx isoforms. For GJs formed of Cx36, there is a distinct aspect to their modulation by intracellular free Mg^2+^ (Palacios-Prado et al., 2013; Palacios-Prado et al., 2014). Using exogenous Cx expression in communication-deficient HeLa cells, junctional conductance, g_j_, between cells expressing Cx36 was shown to increase robustly, several fold when the intracellular concentration of free Mg^2+^ ions, [Mg^2+^]_i_, was reduced from resting levels, which are typically ∼ 1mM. This effect on g_j_ has not been observed in other Cxs (Palacios-Prado et al., 2013). Conversely, when [Mg^2+^]_i_ was increased from resting levels, reductions in g_j_ occurred. However, reductions in g_j_ upon elevating intracellular Mg^2+^ occur in GJs formed of other Cxs suggesting that Cx36 GJ channels exhibit a substantial shift in Mg^2+^ sensitivity such that changes in [Mg^2+^]_i_ at or about physiological levels can robustly impact junctional coupling. In rat brain slices, electrical synapses between neurons of the trigeminal mesencephalic nucleus, and between interneurons from the thalamic reticular nucleus, which are formed of Cx36, also were shown to exhibit sensitivity to reductions in [Mg^2+^]_i_ (Palacios-Prado et al., 2013; Palacios-Prado et al., 2014), indicative that this form of modulation occurs in native tissues and is a property that is intrinsic to the Cx36 isoform.

Changes in [Mg^2+^]_i_ can occur under a variety of physiological and pathological conditions. Since the majority of Mg^2+^ ions are bound to ATP, changes in cytoplasmic ATP levels can affect the levels of [Mg^2+^]_i_ (Luthi et al., 1999). Neuronal cytoplasmic ATP levels indeed have been shown to increase with glucose and lactate exposure (Ainscow et al., 2002) and during periods of reduced activity, such as sleep (Dworak et al., 2010). Conversely, cytoplasmic ATP levels were shown to decrease during periods of increased neuronal activity, such as those associated with wake periods and hyperactivity (Dworak et al., 2010). Pathologically, significant reductions in [Mg^2+^]_i_ can occur following traumatic brain injury (Vink et al., 2009) and is a common feature in various neurological disorders, such as epilepsy, Parkinson’s and Alzheimer’s disease (Barbiroli et al., 1999; Andrasi et al., 2000; Fang et al., 2010). Elevation of [Mg^2+^]_i_ can occur in the brains of patients that have undergone ischemic events and that have been diagnosed with schizophrenia (Hinsberger et al., 1997) or mitochondrial disorders (Barbiroli et al., 1999). In the pancreas, deficiencies in Mg^2+^ levels have been associated with Type 2 diabetes mellitus (Barbagallo and Dominguez, 2007; Ramadass et al., 2015) and pancreatitis (Papazachariou et al., 2000). Thus, the robust sensitivity of Cx36 GJs to intracellular Mg^2+^ can have a large impact on neuronal cell function through modulation of electrical coupling, as well as the communication of signaling molecules and metabolites.

Using a chimeric approach that swapped domains between Cx36 and Cx43, the higher sensitivity of Cx36 GJs to [Mg^2+^]_i_ was attributed, at least in part, to the first extracellular loop (E1) domain and in particular a charged residue, D47, predicted to be a pore-lining residue (Palacios-Prado et al., 2014). Subsequent studies, however, indirectly suggested that the N-terminal (NT) domain also may play a role in Mg^2+^ sensitivity of Cx36 GJs based on the observation that the effects of intracellular pH and [Mg^2+^]_i_ were interdependent (Rimkute et al., 2018). Acidification, which typically produces a robust reduction in g_j_ for most GJs, uniquely produces an increase in g_j_ for Cx36 GJs (González-Nieto et al., 2008). However, when [Mg^2+^]_i_ is reduced, the increase in g_j_ no longer occurs suggesting, perhaps, that pH alters the binding of Mg^2+^ leading to differential effects of acidification on g_j_ depending on the level of [Mg^2+^]_i_. Molecularly, glutamate residues E8 and E12 in the NT domain were shown to play a role in this interdependence between pH and Mg^2+^, although only E8 appeared to impact the increase in g_j_ observed upon reduction of [Mg^2+^]_i_ levels (Rimkute et al., 2018).

The aim of the present study was to more extensively examine the role of the NT domain in the modulation of Cx36 GJ channels by [Mg^2+^]_i_. We examined point mutations at 5 positions, E3, T5, E8, A13 and H18 in the NT domain of Cx36 that, based on homology modeling using the more recent Cx46/Cx50 structure (Myers et al., 2018), are predicted to be exposed to the pore. In comparison to Cx43, which was used in the chimeric approach to examine the molecular determinants of Mg^2+^ regulation, two of the six positions differ in charge, E8 in Cx36 vs G8 in Cx43, and A13 in Cx36 vs K13 in Cx43. We find that charge substitutions at these two positions significantly altered the response of Cx36 to [Mg^2+^]_i_, as did charge substitution at H18. E8Q and H18K substitutions abolished the increase in g_j_ upon exposure to low [Mg^2+^]_i_ and A13K completely abolished the effects of Mg^2+^ at all concentrations. Charge substitutions at the other positions had no effect. Combining electrophysiological recordings that compared the extent as well as the rate of the Mg^2+^ effect on g_j_ in all the mutants, along with measurements of Mg^2+^ permeability using a fluorescent indicator, we developed mathematical models to assess potential mechanisms of Mg^2+^ modulation of Cx36 GJ channels. These models were based on different kinetic schemes of Mg^2+^-binding and the putative effect of Mg^2+^ on the closing each of the component hemichannels in a Cx36 GJ channel. We found that Mg^2+^ most likely acts by binding to and stabilizing the closed conformation of a Cx36 hemichannel and that NT residues, which are at the cytoplasmic entrance of the pore, act electrostatically and allosterically to alter the permeability and/or binding of Mg^2+^postulated to be deeper in the pore in the E1 domain.

## Materials and Methods

### Cells and Culture Conditions

Electrophysiological measurements were performed in RIN cells (rat beta-cell insulinoma, ATCC CRL-2057) transfected with WT or mutant variants of mouse Cx36 fused with enhanced green fluorescent protein (EGFP) attached to the carboxy terminus. Cells were cultured in RPMI 1640 medium, with L-glutamine, supplemented with 10% fetal bovine serum, 1% streptomycin, and 1% penicillin. Mutants of Cx36 were generated using the QuikChange Multi Site-directed mutagenesis kit (Agilent). These mutants were subcloned into pEGFP-N1 vector (Clontech, United States) and transfected into RIN cells using Lipofectamine 2000 (Life technologies, United States). All experiments were performed with stable cell lines.

### *In vitro* Electrophysiological Measurements

Electrophysiological recordings and fluorescence imaging were performed on cells grown on glass coverslips and transferred to an experimental chamber placed on the stage of an inverted microscope Olympus IX70 (Olympus, Japan) equipped with a fluorescence imaging system and a constant flow-through perfusion. Junctional conductance, g_j_, was measured in cell pairs using the dual whole-cell patch clamp technique with EPC-7 (*HEKA*) or EPC-8 (*HEKA*) patch clamp amplifiers. Voltage and current signals were digitized using an A/D converter (National Instrument) and acquired and analyzed using custom-made software. Transjunctional voltages (V_j_) were generated by stepping the voltage in cell-1 while keeping the voltage in cell-2 constant. Junctional current (I_j_) was measured as the current change in cell-2. Patch pipette resistance was maintained below 3 MΩ in order to minimize the influence of series resistance on measurements of g_j_. Recording pipettes were pulled from borosilicate glass capillary tubes with filaments using P-97 micropipette puller (Sutter Instrument Co., United States).

During recording, cells were perfused with an extracellular solution containing (in mM): 140 NaCl, 4 KCl, 2 CaCl_2_, 1 MgCl_2_, 2 CsCl, 1 BaCl_2_, 5 HEPES, 5 glucose, 2 pyruvate, pH 7.4. Patch pipettes were filled with a solution containing (in mM): 130 CsCl, 10 NaAsp, 0.26 CaCl_2_, 5 HEPES, 2 BAPTA, 1 MgCl_2_, pH 7.3. To study the effect of [Mg^2+^]_i_ pipette solutions contained different concentrations of MgCl_2_: 0.01, 1, or 5 mM. To chelate free Mg^2+^ ions in the cell we used 2 mM K_2_ATP in the pipette solution with 0.01 mM Mg^2+^. Maxchelator software was used to calculate free Mg^2+^ ionic concentrations. Solutions were adjusted for differences in osmolarity with the appropriate concentrations of CsCl.

### Fluorescence Imaging Studies

Fluorescence imaging was accomplished using an ORCA digital camera (Hamamatsu) and UltraVIEW (PerkinElmerLifeSciences, Boston, MA, United States) software. We used an excitation filter of 485, and emission filter of 530 to identify cell pairs expressing WT and mutant variants of Cx36. The same filters were used for fluorescence measurement of Mag-Fluo-4. For magnesium transfer studies, a Mg^2+^ fluorescent indicator Mag-Fluo-4 (50 μM) was introduced into cell-1 of a cell pair through a patch pipette with modified standard pipette solution without MgCl_2_. Cell-2 was patched with the pipette containing standard pipette solution and 5 or 10 mM MgCl_2_. After breaking into cell-1, the fluorescence intensity (FI_1_) of Mag-Flou-4 rose to a steady state value. The patch in cell-2 was then opened and the changes in FI_1_ in cell-1 and g_j_ were measured simultaneously. To measure g_j_, repeated, small amplitude voltage ramps (±10 mV) were applied.

### Homology Structure Modeling

Structural homology models of Cx36 and its mutants were built using as a template the structure of a Cx46/Cx50 GJ channel (6MHQ) obtained by cryo-EM (Myers et al., 2018). Homology modeling was carried out using MODELLER. The sequence of Cx36 was aligned with that of Cx46 using MODELLER software and showed a sequence identity of >53%. The best model was selected according to the discrete optimized protein energy score. Input files for building the Cx36 homology model using MODELLER software are provided in Supplementary Material.

### Simulation of the Electrostatic Potential

The electrostatic potentials of all the atoms in the protein were estimated using Adaptive Poisson–Boltzman solver (APBS). To convert PDB files into PQR as input to APBS, we used PDB2PQR server with a PARSE forcefield (Dolinsky et al., 2004). PROPKA was used to assign protonation states at pH 7.3. Dielectric constants were set to 2.0 for the interior (protein) and 80.0 for the exterior (solvent) regions. To calculate electrostatic potentials at a particular location, data from the DX file of the corresponding model were extracted and analyzed using custom-made software written in C# programming language. The visualization was done using Visual Molecular Dynamics and UCSF Chimera software.

### Minimization and Equilibration

Introducing mutations adds features that may not be structurally accurate. To improve the accuracy of the system structure, we performed minimization and equilibration using GROMACS simulation software. The protein was inserted in a lipid bilayer membrane environment using the CHARMM-GUI membrane builder (Wu et al., 2014; Lee et al., 2018). The inner and outer leaflets of the membrane consisted of mixed lipids: phospholipid (PA, PC, PE, PI, and PS) (Locke and Harris, 2009), sphingolipids (SSM and LSM), and sterols (Cholesterol) (Cascio, 2005). The assembled systems were solvated with a sufficient number of TIP3P water molecules. To neutralize the systems, 0.15 M NaCl solution was added. Temperature and pressure were maintained at 303.15 K (30°C) and 1 bar, respectively. The system energy was minimized using the steepest descent algorithm until the maximum force went below 1000 kJ/mol/nm. Each system was equilibrated using input files generated by CHARMM-GUI. To ensure a valid equilibration, each system was verified through investigating the root-mean-square deviation.

### Mathematical Models of Mg^2+^-Induced GJ Channel Gating and Ion Permeability

#### Magnesium Binding and Channel Closure

We developed a series of different mathematical models to examine possible mechanisms of Mg^2+^ action on GJ channels formed of Cx36. All the models were based on the assumption, that Mg^2+^ can bind to each of the apposing hemichannels and that each hemichannel can be described by a linear 3-state reaction scheme of the following form:


$$O\,\begin{array}{c} k_{1}\\ ⇄\\ k_{2} \end{array}\,X\,\begin{array}{c} k_{3}\\ ⇄\\ k_{4} \end{array}\,C.$$

Here, *O* and *C* denote open and closed states of a hemichannel, respectively. *X* denotes an intermediate state, which, could be either open or closed (for simplicity, we did not consider any intermediate values of conductance for this state of the hemichannel). In addition, we presumed that *O*→*X* and *X*→*C* transitions could be either Mg-independent or could reflect the binding of Mg^2+^ ions according to the law of mass action. This gives us eight different models to consider, depending on the assumptions about the intermediate state *X*, as well as *O*→*X* and *X*→*C* transitions (see Table 1).

**TABLE 1:** Different variants of a basic 3-state linear kinetic scheme *O*↔*X*↔*C* to describe Mg^2+^-mediated closure of Cx36 hemichannels.

Model	*O*→*X*	*X*	*X*→*C*
*Model 1*	*Mg^2+^ binding*	*open*	*Mg^2+^ binding*
*Model 2*	*Mg^2+^ binding*	*open*	*Mg-independent*
*Model 3*	*Mg^2+^ binding*	*closed*	*Mg^2+^ binding*
*Model 4*	*Mg^2+^ binding*	*closed*	*Mg-independent*
*Discarded* ^×^	*Mg-independent*	*open*	*Mg^2+^ binding*
Discarded *	*Mg-independent*	*open*	*Mg-independent*
*Model 5*	*Mg-independent*	*closed*	*Mg^2+^ binding*
Discarded *	*Mg-independent*	*closed*	*Mg-independent*

*–Does not contain Mg-dependent transitions.

*X–Redundant Mg-independent transition between two open states.*

As is noted in Table 1, we discarded two models which did not contain any Mg^2+^ ion binding (denoted by *), and another model (denoted by ^×^), which exhibits a redundant Mg-independent transition between two open states. This leaves us with five basic models, each describing somewhat different mechanistic scenarios. For example, in Model 1, a Mg^2+^ binding event occurs in a hemichannel in its open conformation, which remains open until a subsequent binding event occurs resulting in hemichannel closure, whereas in Model 5, a Mg^2+^ binding event occurs in a hemichannel in its closed conformation, with closure achieved through a Mg^2+^-independent gating event. For each of the five basic models, in addition to Mg^2+^ binding according to the law of mass action, we considered the Hill equation to describe Mg^2+^ binding, which can be interpreted as simultaneous binding of *n* Mg^2+^ ions, or more generally, expresses cooperative (*n* > 1), competitive (*n* < 1) or independent (*n* = 1) binding (Yifrach, 2004). This consideration extended our analyses to a total of 10 models, with the number of free parameters varying between five and eight. Model testing was accomplished by fitting a collection of experimental data on WT and variant Cx36 GJs. The numerical model fitting experiments did not permit distinction among more complex models due to high parameter uncertainty and overfitting.

#### Intracellular Concentration of Mg^2+^ Ions

The flux of Mg^2+^ ions through GJ channels was simulated in the same way for all models. Because g_j_ values of cell pairs expressing WT and mutant variants of Cx36 were typically low, Mg^2+^ flux was relatively low and the Mg^2+^ ion concentration of the donor cell could be presumed to remain constant and equal to the value in the patch pipette throughout the duration of the experiments. For modeling, we presumed that the rate of diffusion of Mg^2+^ ions was proportional to the concentration difference between the cells as described by the Fick’s law, which can be described by the following system of ordinary differential equations (ODEs):


$$\left\{ \begin{array}{c} \frac{d\,{\left[M\,g^{2+}\right]}_{1}}{d\,t}=-P\cdot g_{\text{j}}\cdot \left({\left[M\,g^{2+}\right]}_{2}-{\left[M\,g^{2+}\right]}_{1}\right)\\ \frac{d\,{\left[M\,g^{2+}\right]}_{2}}{d\,t}=-P\cdot g_{\text{j}}\cdot \left({\left[M\,g^{2+}\right]}_{1}-{\left[M\,g^{2+}\right]}_{2}\right) \end{array}\right.$$

Here, *i* (*i* = 1,2) denotes the hemichannel in each of two apposing cells, cell-1 and cell-2. The constant *P* quantifies the permeability of a GJ channel to Mg^2+^ ions and *g*_j_ (0 ≤ *g*_j_ ≤ 1) is normalized junctional conductance, which can also be interpreted as the proportion of open channels between two cells. Because the inspected RIN cells were of similar sizes we made the simplifying assumption that both cells of a pair were of the same volume. Any differences in cell volumes should average out in different experiments and should not distort modeling results to any significant degree. In addition, to simulate experiments with highly asymmetric concentrations of Mg^2+^ in the pipette solutions, we subtracted a component *P*_*leak*_⋅[*Mg*^2 +^]_i_ to account for the leak of Mg^2+^ into the patch pipette in the recipient cell. The magnitude of this leak current parameter *P*_*leak*_ was estimated as described in (Palacios-Prado et al., 2013).

#### Model Fitting and Parameter Estimation

Parameter estimation was performed using the MATLAB global optimization toolbox. To estimate model parameters, the simulated changes in g_j_ and intracellular Mg^2+^ over time were fit simultaneously to averaged electrophysiological and fluorescent imaging data for WT Cx36 and each variant. That is, we assumed that WT Cx36 and each of the variants can exhibit a distinct set of model parameters, which describes its Mg^2+^ binding affinity, permeability, etc. To solve a system of ODEs we applied the Euler method; the integration step was chosen to be sufficiently small (in 0.001–0.00001 range) to ensure the stability of the calculations. To avoid parameter redundancy and overfitting, we presumed fluorescence intensity of the Mg^2+^ indicator was proportional to the intracellular Mg^2+^ concentration and the constant of proportionality should be reflected in parameter *P*, when simulated Mg^2+^ concentration curves were fitted directly to measured changes in fluorescence intensity over time.

We applied the method of least squares for model fitting. That is, global optimization was performed to obtain model parameters which minimized the sum of squared errors (SSE):


$$S\,S\,E=\sum_{i=1}^{n}{\left(y_{i}-{\hat{y}}_{\text{i}}\right)}^{2}.$$

Here *y*_*i*_ denotes data points obtained from the experiments while ${\hat{y}}_{\text{i}}$ denotes the respective values predicted by a model; *n* is total number of experimentally observed values.

In theory, inclusion of an additional parameter into a model can only result in a lower SSE during the model fitting, even if the addition carries little or no relevance. Thus, to avoid such overfitting we used the Akaike Information Criterion (AIC). For least squares model fitting problems, AIC can be estimated from the following equation:


A⁢I⁢C=2⁢(k+1)+n⋅l⁢n⁢(S⁢S⁢E)-n⋅l⁢n⁢(n).

Here *k* denotes the number of model parameters.

The value of AIC for a given model is useful only in comparison to AIC values of other models. That is, a model with the minimum value of AIC relative to all the others is considered the best. An increase in the number of model parameters raises the AIC value. Thus, such an addition is only justified if it significantly reduces the value of SSE. In this way, the use of AIC for model selection gives a preference for a more parsimonious model. In addition, it allows for a direct comparison among models which have different numbers of parameters.

If two models are applied for the same experimental dataset, they can only differ in the values of the delta AIC (ΔAIC), which is defined as follows:


Δ⁢A⁢I⁢C=2⁢k+n⋅l⁢n⁢(S⁢S⁢E).

As a rule of thumb, two candidate models are considered significantly different in fitting ability if the difference in their ΔAIC values exceeds 2 (Symonds and Moussalli, 2011).

To compare different models we used the Akaike weights (*w*_i_), which are defined as follows:


$$w_{i}=\frac{\text{exp}\,\left(-\Delta \,A\,I\,C_{i}/2\right)}{\sum_{j=1}^{m}\exp\left(-\Delta \,A\,I\,C_{i}/2\right)}.$$

Here Δ*AIC*_*i*_ denotes the value of ΔAIC of a respective model and m is the number of candidate models. The Akaike weight can be interpreted as the probability that the respective candidate model is the best among all the candidate models.

### Data Analysis

The analysis and statistics were performed using RStudio and custom-made software written in C# or Visual Basic for Applications languages. Statistical analyses were performed using an unpaired Student t-test. Differences were considered statistically significant at *p* < 0.05.

## Results

### Charge Substitutions in the NT Domain Can Significantly Alter Regulation of Cx36 GJs by Mg^2+^

**FIGURE 1:**
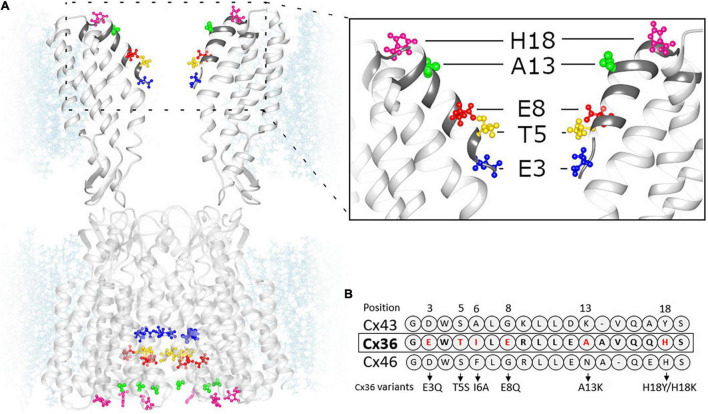
Homology model of Cx36 GJ channel structure. **(A)** Cx36 GJ channel based on crystal structure of Cx46 (6MHQ) following energy minimization and equilibration. (Inset) The segment of GJ channel corresponding to NT domain (dark gray). The color-coded residues of Cx36 were replaced by the residues presented in panel **(B)**. **(B)** Sequence alignment of Cx43, Cx36 and Cx46 NT domains.

The predicted structure of the Cx36 GJ channel based on homology modeling using the Cx46/Cx50 cryo-EM structure is shown in Figure 1A. Given that the molecular basis for the unique Mg^2+^ regulation of Cx36 was investigated by swapping domains with Cx43, Figure 1B includes the sequence alignment for the NT domain of Cx43 along with Cx36 and Cx46. Positions 3, 5, 8, 13, and 18 in the NT domain of Cx36 are predicted to be exposed to the pore with A13 and H18 located at the wide cytoplasmic entrance of the pore vestibule. We individually mutated each of these residues and examined the effects on regulation by the intracellular Mg^2+^ ion concentration. To assess regulation by intracellular Mg^2+^, we measured g_j_ in pairs of cells expressing WT or variants of Cx36 with three different concentrations of Mg^2+^ in the patch pipettes, 0.01, 1.0, and 5.0 mM. At each Mg^2+^ concentration we measured the change in g_j_ over time, noting the initial value upon establishing a dual whole cell recording, (g_j,init_) and the final steady-state value (g_j,ss_). An index of the effect of Mg^2+^ was given by the ratio g_j,ss_/g_j,init_.

**FIGURE 2:**
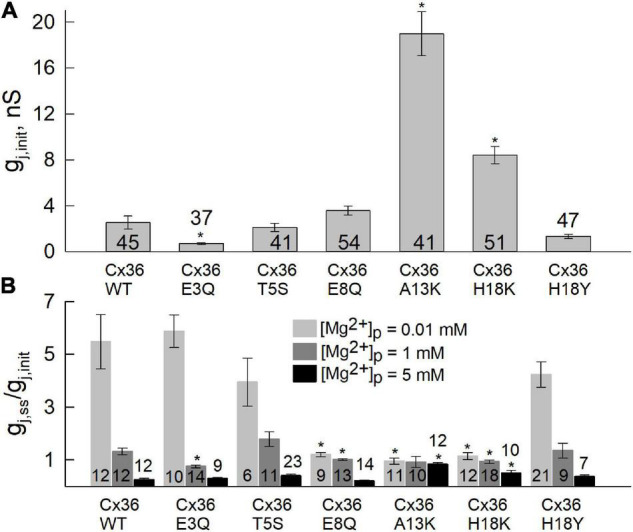
Differences in sensitivity to [Mg^2+^]_p_ between Cx36 WT and NT variants. Experiments were performed in pairs of RIN cells expressing Cx36 WT and NT variants, tagged with EGFP. **(A)** Initial junctional conductance (g_j,init_) was registered immediately after the patch was opened. **(B)** Average g_j_ value of Cx36 and mutants at the steady-state level, normalized to initial conductance, g_j,ss_/g_j,init_, recorded using pipette solutions with 0.01, 1, or 5 mM [Mg^2+^]_*p*_. Statistically significant (*p* < 0.05) differences of each mutant compared to WT Cx36 are indicated by asterisks. Data were obtained by applying repeated small voltage ramps from -10 to 10 mV. Error bars correspond to standard errors, and total numbers of experiments are indicated within or above the columns.

Results for each of the substituted variants at positions, 3, 5, 8, 13, and 18, are summarized in Figure 2. Plots are shown for mean values of g_j,init_ (Figure 2A) and g_j,ss_/g_j,init_ (Figure 2B). Considering the two negatively charged putative pore-lining Glu residues at positions, 3 and 8, E3 is conserved in charge compared to Cx43, whereas E8 is a neutral Gly residue. We neutralized each of these positions by replacing Glu with Gln. E3Q GJ channels behaved similar to WT Cx36 GJ channels, except for small, albeit significant reduction in g_j,ss_/g_j,init_ that was accompanied by a lower mean value of g_j,init_. The E8Q mutation, however, essentially abolished the increase in g_j_ at low [Mg^2+^]_i_, but retained sensitivity to high [Mg^2+^]_i_. The mean value for g_j,init_ was not significantly different from WT Cx36.

Position 13, which is an Ala residue in Cx36, has a corresponding positively charged Lys in Cx43. Thus, we replaced A13 with Lys. The A13K mutation resulted in a complete loss of the response to all applied concentrations of Mg^2+^ (Figure 2B). Correspondingly, the A13K mutation showed a robust increase in the mean value of g_j,init_. Moving further out toward the cytoplasmic vestibule, we replaced H18 with Lys which also resulted in significantly reduced sensitivity to intracellular Mg^2+^. Like A13K, H18K also exhibited a significantly higher mean value of g_j,init_ compared with WT Cx36 (Figure 2A). Neutral-to-neutral substitutions at positions 5, and 18, T5S and H18Y, did not show any significant differences in sensitivity to intracellular Mg^2+^ compared to WT Cx36. An I6A mutation resulted in lost channel function (data not shown) consistent with the roles of non-pore-lining residues in the maintenance of NT structure (Purnick et al., 2000a). Thus, overall, the results suggest that alterations in charge in NT residues can significantly affect sensitivity of Cx36 GJs to intracellular Mg^2+^.

### Evaluation of Mg^2+^ Permeability and Changes in g_j_ in WT and NT Mutant Cx36 GJ Channels

Given that the NT domains constitute the cytoplasmic vestibules of a GJ channel pore, we wanted to evaluate whether the NT substitutions, particularly those that affected regulation by Mg^2+^, affected Mg^2+^ permeation. To assess permeability of Mg^2+^ through WT, E3Q, E8Q, A13K and H18K Cx36 GJ channels, we used a cell-impermeant fluorescent Mg^2+^ indicator Mag-Fluo-4 (MF4). Experiments were performed using the same protocol as described in Palacios-Prado et al. (2013) in which we first obtained a whole-cell recording with a Mag-Fluo-4-containing pipette on one cell (cell-1) and then introduced Mg^2+^ in the other cell (cell-2) by establishing a second whole-cell recording with a Mg^2+^-containing pipette. This allowed simultaneous measurement of g_j_ and changes in Mag-Fluo-4 fluorescence arising from the flux of Mg^2+^ through GJ channels. The effects of Mg^2+^ occurred over a time course of a few minutes during which time we saw no measurable changes in plaque size.

**FIGURE 3:**
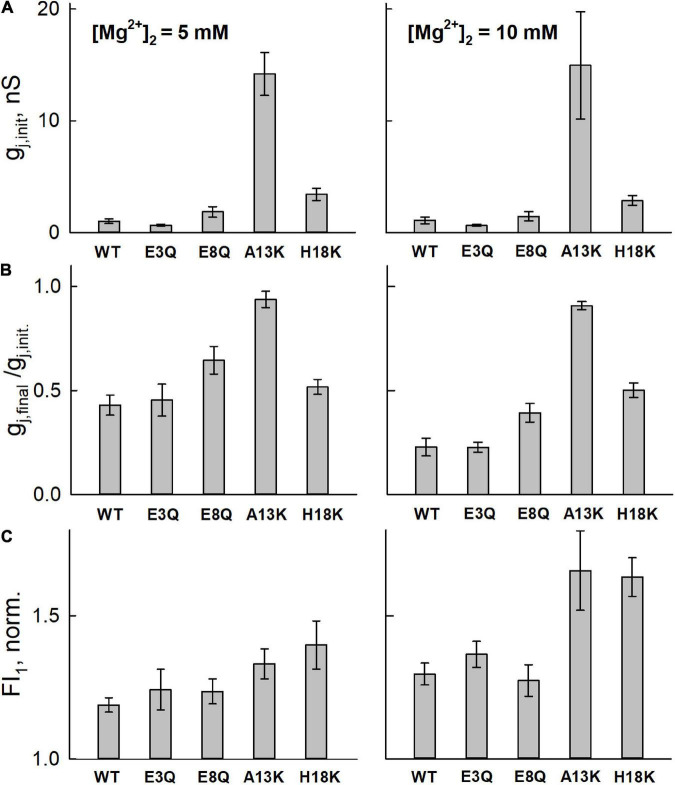
Averaged data from simultaneous electrophysiological and fluorescence recordings in RIN cells expressing WT or mutated Cx36 GJ channels. **(A)** Bar graphs of initial g_j_ values (g_j,init_), measured upon establishing a whole-cell patch recording in the second cell of a pair (cell-2) with a pipette containing high intracellular Mg^2+^. Thus, g_j,init_ is a measure of g_j_ prior to any appreciable changes in intracellular Mg^2+^. **(B)** Mean g_j_ (g_j_,_*final*_) assessed at the end of a 5 minute time interval normalized to initial value (g_j_,_init_) assessed at the time a whole cell recording was established in cell-2. **(C)** Measurement of the change in Mag-Fluor-4 fluorescence in the same recipient cells (cell-1; FI_1_) at the end of the 5 min time interval. FI_1_ was normalized to the initial value approximating the resting Mg^2+^ level. All experiments were performed with pipette-2 containing 5 mM (first column) or 10 mM (second column) Mg^2+^. Values represent the mean and standard error; *n* = 6 for 5 mM Mg^2+^ and *n* = 5 for 10 mM Mg^2+^.

**FIGURE 4:**
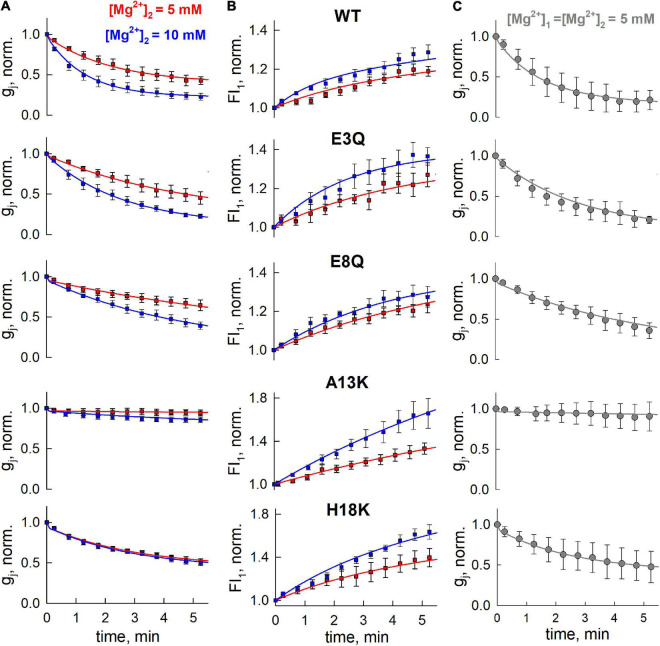
Model fitting to data from simultaneous electrophysiological and fluorescence recordings. **(A,B)** Solid lines are fits to the electrophysiological **(A)** and fluorescence imaging **(B)** data presented in Figure 3 using Model 5 with Hill equation. **(C)** Reductions in g_j_ recorded under symmetric Mg^2+^ conditions (both pipettes contained 5 mM Mg^2+^. Error bars denote standard deviations (*n* = 7). Data in panel **(C)** was not used in the fits to obtain parameters, but only for validation of the model.

We used two concentrations of Mg^2+^ in the patch pipette for cell-2, 5 and 10 mM. The mean initial g_j_ values (Figure 3A) were typically low in cell pairs expressing WT Cx36, E3Q, and E8Q, whereas they were higher in those expressing A13K and H18K, consistent with results from experiments summarized in Figure 2 under symmetric Mg^2+^ conditions. Changes in g_j_ and Mag-Fluo-4 fluorescence at the end of a 5 min interval are shown in Figures 3B,C, respectively. To permit the assessment of mean values, for each cell pair, the data were normalized to the initial values of g_j_, g_j,final_/g_j,init_, and the initial fluorescence in cell-1. Changes in g_j_ and fluorescence were followed after establishment of a whole-cell patch recording in cell-2. Again, consistent with results in Figure 2, all the variants, except for A13K, exhibited reductions in g_j_ when high Mg^2+^ was introduced into the cytoplasm of cell-2. The magnitudes of the changes in g_j,final_/g_j,init_ for E3Q and E8Q GJs were similar to WT Cx36, although both variants, especially E8Q, exhibited somewhat slower kinetics compared to WT (see Figure 4). Also, g_j_ decreased significantly more when the pipette contained 10 mM Mg^2+^ compared to 5 mM Mg^2+^. H18K, although exhibiting sensitivity to high Mg^2+^, showed no difference between 5 and 10 mM Mg^2+^. For these same cell pairs, the measured Mag-Fluo-4 fluorescence showed similar changes for E3Q and E8Q compared to WT. A13K and H18K GJs showed somewhat larger increases in fluorescence, FI_1_, but g_j,init_ in these cell pairs was significantly higher suggesting a substantially reduced permeability to Mg^2+^ when normalized to conductance. In the next section, we take all these data into account and apply mathematical models to gain insights into mechanisms of Mg^2+^ action.

### Fitting Mathematical Models to Electrophysiological and Fluorescent Imaging Data

For model fitting, we used the 10 different 3-state models detailed in Methods. These models were fit to the electrophysiological and fluorescence imaging data presented in Figures 3A,C using global optimization. For independent validation, we examined how well the obtained parameters could explain the reductions in g_j_ for WT and mutant variants of Cx36 recorded from separate data sets that measured the changes in g_j_ over time under different conditions, namely both pipettes containing high Mg^2+^ (symmetric 5 mM [Mg^2+^]_p_). Table 2 presents the values of summary ΔAIC and Akaike weights for model fitting data, as well as SSEs for independent validation (ΔAIC and SSE values grouped by Cx36 variants are presented in Supplementary Material).

**TABLE 2:** The fit and independent validation measures of different models applied to electrophysiological and fluorescent imaging data.

Model	Model fitting		Validation	Model	Model fitting		Validation
	ΔAIC*_i_*	*w*_i_	SSE		ΔAIC*_i_*	*w*_i_	SSE
Model 1	–901.55	0.0000	0.0879	Model 1, Hill	–902.51	0.0000	0.1017
Model 2	–972.53	0.0000	0.1336	Model 2, Hill	–941.33	0.0000	0.0552
Model 3	–920.01	0.0000	0.0838	Model 3, Hill	–914.52	0.0000	0.0869
Model 4	–912.43	0.0000	0.1216	Model 4, Hill	–1009.95	0.3432	0.0504
Model 5	–993.96	0.0001	0.0669	Model 5, Hill	–1011.25	0.6568	0.0470

Data in Table 2 shows that that Model 5 with the Hill equation for Mg^2+^ binding provided the best fit. The difference between ΔAICs of the best and second best model (Model 4 with the Hill equation) exceeds 2, which is considered significant. The ratio of the respective Akaike weights (∼1.91) indicates that Model 5 with the Hill equation has approximately a two times higher probability of being better than Model 4 with the Hill equation and is the best among all the considered models. In addition, Model 5 with the Hill equation provided the lowest SSE for independent validation data, which further strengthens our confidence that it is the most suitable candidate model.

The kinetic scheme of this model (Model 5, Hill) is presented below:


$$O\,\begin{array}{c} k_{1}\\ ⇄\\ k_{2} \end{array}\,C$$

$$C+n\cdot M\,g^{2+}\,\begin{array}{c} k_{3}\\ ⇄\\ k_{4} \end{array}\,{\text{C-Mg}}^{2+}$$

The first transition of this model is Mg-independent, which can be interpreted as stochastic gating of a hemichannel that is V_j_-dependent. The second transition can be interpreted as the binding of Mg^2+^ ion(s) to the closed state of a hemichannel and subsequent stabilization of this closed state. The system of ODEs, describing Mg^2+^-binding and closure of a single hemichannel is presented below:


$$\left\{ \begin{array}{c} \frac{d\,{\left[O\right]}_{i}}{d\,t}=-k_{1}\cdot {\left[O\right]}_{i}+k_{2}\cdot {\left[C\right]}_{i}\\ \frac{d\,{\left[C\right]}_{i}}{d\,t}=k_{1}\cdot {\left[O\right]}_{i}-\left(k_{2}+k_{3}\cdot {\left[M\,g^{2+}\right]}_{i}^{n}\right)\cdot {\left[C\right]}_{i}+k_{4}\cdot {\left[C-M\,g^{2+}\right]}_{i}\\ \frac{d\,{\left[C-M\,g^{2+}\right]}_{i}}{d\,t}=k_{3}\cdot {\left[C\right]}_{i}\cdot {\left[M\,g^{2+}\right]}_{i}^{n}-k_{4}\cdot {\left[C-M\,g^{2+}\right]}_{i} \end{array}\right.$$

Here, *i* (*i* = 1,2) denotes the hemichannel in each of two apposing cells, cell-1 or cell-2 and *n* is the Hill coefficient for the binding of Mg^2+^ ions. State variables [*O*]_*i*_, [*C*]_*i*_ and [*C*−*Mg*^2 +^]_*i*_ each denotes the probability that a hemichannel resides in an open, closed or Mg^2+^-stabilized closed state, respectively. These variables must be in the range [0;1] and satisfy the following conservation rule:


$${\left[O\right]}_{i}+{\left[C\right]}_{i}+{\left[C-M\,g^{2+}\right]}_{i}=1.$$

Based on the assumption that each apposing hemichannel can close the GJ channel pore, g_j_ can be expressed as *g*_*j*_ = [*O*]_1_⋅[*O*]_2_. The estimated parameters of this model for WT and mutated Cx36 channels are presented in Table 3. The kinetic schemes and estimated parameters obtained from other considered models are presented in Supplementary Material.

**TABLE 3:** The estimated parameters of Model 5 with Hill equation.

Cx	*k*_1_, (min^–1^)	*k*_2_, (min^–1^)	*k*_3_, (min⋅mM^–1^)	*k*_4_, (min^–1^)	*n*	*P*, (min⋅nS^–1^)
WT	5.35	386.57	3.2770	0.1802	1.12	0.0135
E3Q	3.52	392.28	3.2366	0.0417	1.08	0.0258
E8Q	5.88	271.07	0.7168	0.0197	1.07	0.0076
A13K	3.05	179.34	0.0026	0.0637	2.84	0.0011
H18K	9.80	337.60	3.2391	0.2008	0.11	0.0072

The estimated values of model parameters suggest that the observed slower changes in g_j_ for E3Q and E8Q as compared to WT GJ channels mostly result from a decreased dissociation of Mg^2+^ ions from the putative binding site. As for WT Cx36, the estimated value of the Hill coefficient for both E3Q and E8Q variants was close to 1, which indicates the absence of either cooperative or competitive binding of Mg^2+^ ions. The values of parameter *P* indicate that E3Q exhibited an increased permeability to Mg^2+^ ions whereas E8Q exhibited a decrease compared to WT. The estimated parameters for A13K reflect two main distinguishing features. First, A13K was the only variant that resulted in a substantially reduced Mg^2+^ binding rate, as indicated by a very low value of *k*_3_, or more precisely, the reduced ratio *k*_3_/*k*_4_, compared to WT and the other variants. In addition, the estimated value of *P* indicates that A13K exhibits a more than 10-fold reduction in permeability to Mg^2+^ ions. H18K also exhibited reduced permeability to Mg^2+^, but similar in magnitude to E8Q. Compared to WT Cx36, however, H18K also showed an increase in stochastic gating activity at V_j_ = 0 mV (see larger *k*_1_ value) as well as a much lower value of the Hill coefficient. The latter may indicate a very low cooperativity for Mg^2+^ ion binding and could explain saturation of the Mg^2+^ effects on g_j_ sensitivity at 5 mM. In addition, it would result in a lower sensitivity to high Mg^2+^ concentrations.

The solid lines in Figure 4 represent the fits to the data and show that this model can account for the changes in g_j_ and Mag-Fluo-4 fluorescence (Figures 4A,B). Using the same parameters obtained from these fits, simulated responses (solid lines) fit an independent data set quite well both in terms of the magnitude and the kinetic changes in g_j_ under conditions in which both patch pipettes containing 5 mM Mg^2+^ (Figure 4C).

### Application of the Mathematical Model to Understand and Predict GJ Channel Behavior

**FIGURE 5:**
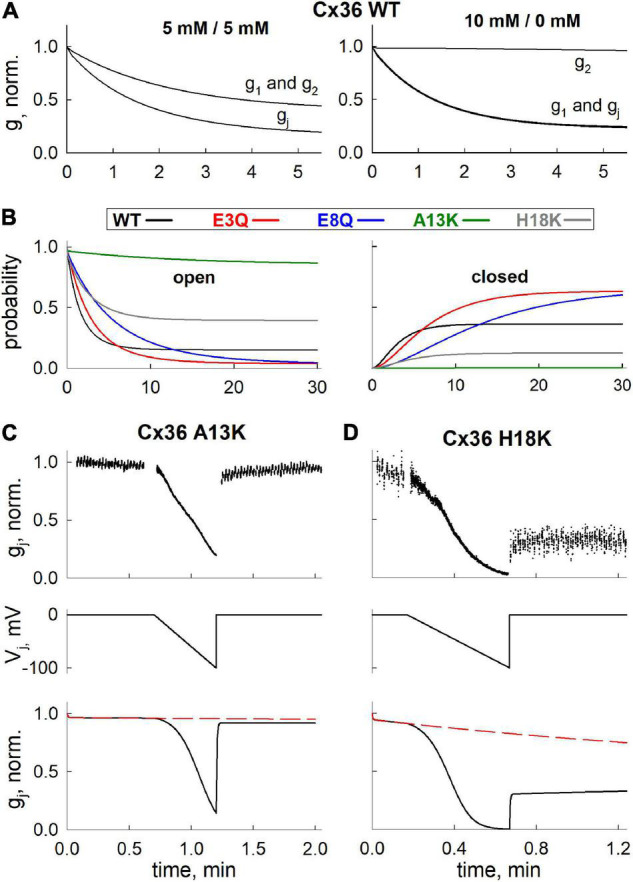
Application of mathematical model to simulate Mg^2+^-induced closure of hemichannels, formed of WT and mutant Cx36. **(A)** Simulated g_j_ decrease of Cx36 WT GJ channels (g_j_) and hemichannels (g_1_ and g_2_) at different levels of [Mg^2+^]_i_. **(B)** Kinetics of open (i.e., both hemichannels are open) and closed (i.e., both hemichannels reside in closed, Mg^2+^-bound state) state probabilities of GJ channels formed of different Cx36 variants, simulated using [Mg^2+^]_1_ = [Mg^2+^]_2_ = 5 mM protocol. **(C,D)** Experimentally measured (upper panels) and simulated (lower panels) g_j_ decrease and recovery after application of V_j_ ramps (middle panels), in A13K and H18K channels. Electrophysiological recordings and simulations were performed using [Mg^2+^]_1_ = [Mg^2+^]_2_ = 5 mM protocol.

We performed computational simulations using parameters derived from the best fit model to assess how the component hemichannels in a GJ channel may be behaving in response to changes in cytoplasmic levels of Mg^2+^, which cannot be directly measured experimentally. As can be seen from our electrophysiological data (Figures 4A,C), the changes in g_j_ obtained using 10 mM Mg^2+^ in one patch pipette were similar to data obtained from experiments in which both patch pipettes contained 5 mM Mg^2+^. These data suggest a mechanism that allows for both hemichannels to be closed upon exposure to Mg^2+^ ions from their respective cytoplasmic compartments. Figure 5A shows simulated changes in g_j_ as well as the conductances of the separate hemichannels (g_1_ and g_2_) over a 5 min time frame. Under symmetric 5 mM Mg^2+^conditions (left panel), both hemichannels are predicted to exhibit the same reductions in conductance, which results in a decrease in g_j_ reflecting the combined closed probabilities of the hemichannels. Conversely, with 10 mM Mg^2+^ only on one side, the reduction in g_j_ essentially reflects closure of the hemichannel on the high Mg^2+^ side, but resulted in a similar overall change in g_j_ as simulated with symmetric 5 mM Mg^2+^.

We also used the model to predict the changes in open and closed GJ channel probabilities under symmetric 5 mM Mg^2+^ conditions over a longer time frame of 30 min. The open and closed probabilities plotted are those in which both hemichannels are open (left panel in Figure 5B) or both hemichannels are closed in their Mg^2+^-stabilized closed conformations (right panel in Figure 5B); the latter information would not be directly observable from electrophysiological recordings. As in all simulations, the probabilities reflect the values at V_j_ = 0. For the A13K mutation, the simulation indicates a high maintained open probability of ∼1.0 even when the channel is exposed to 5 mM Mg^2+^. According to the model parameters, this high open channel probability is due to a combination of factors including a low Mg^2+^ permeability and, most importantly, a near absence of Mg^2+^ binding. H18K channels also showed a higher maintained open probability and a reduced probability that both hemichannels reside in a Mg^2+^-stabilized closed conformation. For these channels, however, this is mostly due to a decrease in the Hill coefficient, which resulted in a relatively lower binding rate for Mg^2+^ ions at high [Mg^2+^]_i_ concentrations. Charge substitutions at positions 3 and 8, E3Q and E8Q, resulted in reduced open probabilities and increased probabilities that both hemichannels resided in Mg^2+^-stabilized closed conformations, mainly due to reduced dissociation of Mg^2+^ from its binding site. Although the predicted open and closed probabilities of these mutants were similar at 30 min, they differed in their kinetics, as observed experimentally on a shorter time scale.

Finally, we applied our model fit parameters to see if they can account for an experimentally observed phenomenon in Cx36 GJ channels, which is impeded recovery of g_j_ in the presence of Mg^2+^ following channel closure promoted by applying either V_j_ or chemical stimuli (Palacios-Prado et al., 2013). Using a voltage ramp protocol to promote channel closure, we recorded from H18K and A13K GJs in symmetric 5 mM Mg^2+^ conditions. H18K channels retain some sensitivity to 5 mM Mg^2+^ whereas A13K channels show no sensitivity to Mg^2+^ altogether. Upon application of a voltage ramp, H18K channels exhibited a robust decrease in g_j_. However, upon stepping back to V_j_ = 0, there was a small, fast increase in g_j_ followed by a very slow increase that resembled a plateau over a shorter time interval characteristic of the impeded recovery observed in WT Cx36 (Figure 5D). For A13K channels, which retain the ability to gate closed by V_j_, there is essentially a full and rapid recovery of g_j_ following application of a V_j_ ramp (Figure 5C). Simulations using parameters for these channels obtained from fits to the data in Figure 4 are shown in Figures 5C,D. Because rate constants *k*_1_ and *k*_2_ can be interpreted as opening and closing rates of V_j_-dependent gating, it follows from our previously published V_j_-gating model (Snipas et al., 2020) that the values of *k*_1_ and *k*_2_ of a hemichannel should increase or decrease exponentially with V_j_ (depending on the sign of V_j_ and V_j_-gating polarity of Cx isoform) during the application of V_j_ ramp. The black solid lines (Figures 5C,D) show the predicted changes in g_j_ over time when a V_j_ ramp is applied with the channels exposed to symmetric 5 mM Mg^2+^ conditions. The dashed red lines show the predicted changes in g_j_ in the absence of the V_j_ ramp, reflecting changes that would occur simply upon exposure to symmetric 5 mM Mg^2+^ conditions at V_j_ = 0 (i.e., transition rates *k*_1_ and *k*_2_ remain constant). The simulations reproduced the experimentally-observed behaviors of H18K and A13K GJ channels quite well. According to our model fitting, the impeded recovery in H18K channels is explained by increased entrance into the Mg^2+^-stabilized closed state promoted by the V_j_- induced closure. The rapid and complete recovery of g_j_ for A13K channels can be explained by a lack of Mg^2+^ binding and thus lack of a Mg^2+^-stabilized closed state reflected by an almost 450-fold decrease in the ratio *k*_3_/*k*_4_ compared to WT and the other variants. In addition, the estimated ratios *k*_1_/*k*_2_, which, according to our model, should reflect the intrinsic V_j_ sensitivities of the channels, corresponded well with electrophysiological data. For example, our model predicts that A13K channels should exhibit a comparable V_j_ sensitivity to WT Cx36 channels, while H18K channels should be significantly more V_j_ sensitive. The averaged g_j_-V_j_ curves obtained from voltage ramp experiments recorded at symmetric 5 mM Mg^2+^ conditions (see Supplementary Figure 1) confirm this prediction, thus providing additional support for models in which Mg^2+^ stabilizes hemichannel closure caused by V_j_ gating events.

### Comparison of Electrostatic Potentials Between Wild Type and Mutated Cx36 Using Homology Modeling

**FIGURE 6:**
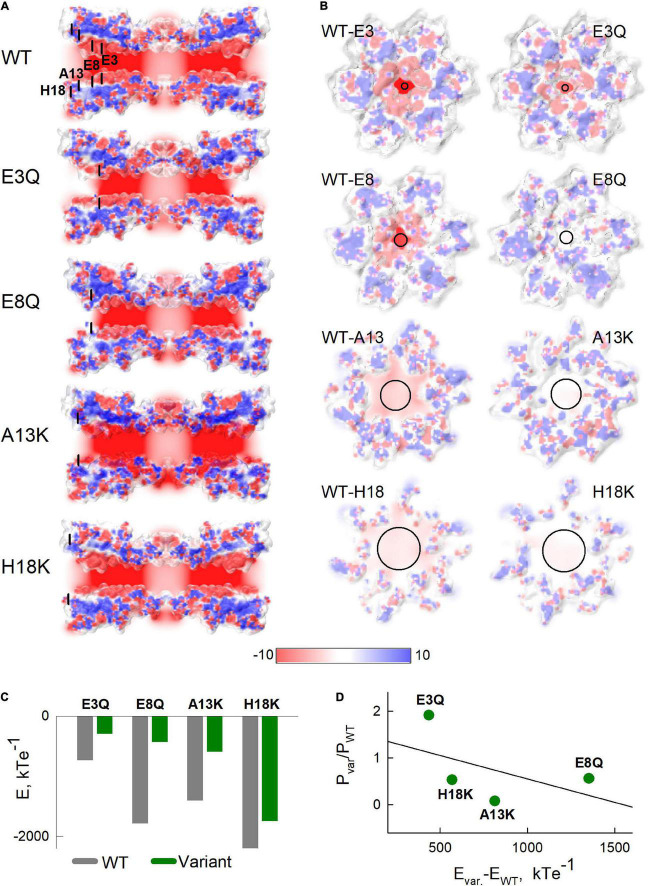
Electrostatic potentials of Cx36 and NT variants. **(A)** Slices along the pore of the Cx36 showing the electrostatic potentials. Black lines indicate locations of mutated residues. **(B)** Cross-sections through the electrostatic potential surfaces of Cx36 WT and mutants in the vicinities of the 3rd, 8th, 13th, and 18th residues viewed from the extracellular side. In both panels **(A,B)**, potentials were scaled between –10 and 10 kTe^–1^ (see color scale below). **(C)** Sums of electrostatic potentials, calculated in the vicinities of the substituted residues. Calculations were performed by summing the average values of electrostatic potentials in 1x1x1 angstrom cubes, located alongside the pore in three layers, closest to the cross-sections presented in panel **(B)**. **(D)** The relationship between the differences in sums of the electrostatic potentials from panel **(C)** and the ratios of permeability coefficients *P*, estimated from Model 5 with Hill equation, for each variant. The black solid line shows a fitted linear regression curve.

Given the effects we observed with charge substitutions in NT and that the NT domain constitutes the cytoplasmic vestibule of the pore, it is possible that charged residues in NT exert an electrostatic effect that reduces Mg^2+^ occupancy inside the pore, thereby affecting access to a binding site proposed to be within the extracellular span of the pore (Palacios-Prado et al., 2014). Thus, we evaluated the electrostatic potentials along and across the pore for WT Cx36 and each of the variants. Figures 6A,B show the corresponding maps of electrostatic potentials. Maps in Figure 6B are presented as viewed from the cytoplasmic side.

The calculated electrostatic potential profiles along the long axis of the channel (Figure 6A) show an overall negative charge bias, which was shifted more positive near the vicinities of substituted residues. Electrostatic potentials viewed in cross-sections of the Cx36 channels at the locations of each of the mutated residues are shown in Figure 6B. Sums of potentials calculated inside these cross-sections of the pore are shown in Figure 6C and suggest that the substitutions resulted in substantial reductions in the negativities of the electrostatic potentials. Figure 6D shows the relationship between the reduction in negative charge inside channel pore and the change of Mg^2+^ permeability evaluated using our mathematical model. The data reveal a moderate negative correlation (estimated correlation coefficient was equal to –0.505) between the shifts in the electrostatic potentials and the respective ratios of the permeability coefficients. The deviations of the data points from the predicted linear relationship between these two variables (solid black line in Figure 6D) suggest that the location along the pore may be a relevant factor. That is, the H18K and A13K substitutions, which are located closest to the cytoplasm, exhibited the more robust reductions in permeability than predicted by the simple linear relationship (data points below the line). In contrast, higher than expected permeabilities (data points above the line) were observed for E8Q and E3Q variants, which are located deeper inside the channel pore.

Another feature, which is visible in Figure 6A, is an alteration in the putative shape of the pore boundary among the different variants. Some of these changes might be due to stochastic calibration of the channel structure when performing homology modeling. However, some of these changes could reflect allosteric reconfiguration of the pore-lining residues. It is likely that such alterations would have an effect on biophysical properties of the channel, such as ion permeability or ligand binding affinities.

Taken together, data using electrostatic calculations show that some of the NT substitutions can affect the surface potential, and possibly, the overall structure of the channel pore. These changes could contribute to a changed balance in ion distribution inside the channel pore, including at the putative Mg^2+^ binding site.

## Discussion

Connexin36 GJs, the principal elements of electrical synapses in the CNS, uniquely exhibit a high sensitivity to intracellular Mg^2+^, which represents a potentially important mechanism for mediating plasticity of electrical synaptic transmission (Palacios-Prado et al., 2013). In this study, we explored the role of the Cx36 NT domain in Mg^2+^ modulation. Although exchange between Cx36 and Cx43 sequences identified the first extracellular loop domain (E1) and the D47 residue in particular, as playing a critical role, the NT domain was subsequently implicated because of an interdependence between Mg^2+^ and pH modulation that was affected by mutations in the NT domain (Rimkute et al., 2018). Here we explicitly examined the role of NT on Mg^2+^ modulation, focusing on putative pore-lining residues spanning the length of the pore funnel, which is formed entirely by the NT domain. In keeping with the chimeric approach that exchanged Cx36 and Cx43 domains, the substitutions at the NT positions we examined were, in part, intended to convert Cx36 to Cx43 sequence. Of the 5 putative pore-lining positions examined, substitutions at 3 positions showed robust effects on Mg^2+^ modulation. These three substitutions, E8Q, A13K, and H18K, all resulted in localized reductions in the negative charge profiles of the pore either by neutralizing a negatively charged residue or changing a neutral residue to a positively charged one. Two of the three substitutions, E8Q and H18K, resulted in GJ channels that retained sensitivity to high intracellular Mg^2+^, but all three substitutions eliminated the robust increase in g_j_ observed in Cx36 GJ channels exposed to low intracellular Mg^2+^ concentrations. These results suggest that E8Q, A13K and H18K substitutions all produce a shift toward reduced apparent affinity for intracellular Mg^2+^. A concomitant increase in g_j_,_init_, the value of g_j_ at the beginning of a recording, for A13K and H18K is consistent with a reduced apparent affinity and thus, reduced inhibition by Mg^2+^ at physiological concentrations. A13K GJ channels were distinguished by insensitivity to high intracellular Mg^2+^ concentrations as well, up to 10 mM, suggesting that A13K produced either a complete loss of Mg^2+^ binding or a very large shift in apparent affinity. Unlike charge substitutions, neutral substitutions did not result in measureable changes in modulation by Mg^2+^. These include neutral H18Y and T5S substitutions; both are substitutions to residues present in Cx43. The one exception was a charge substitution at E3, E3Q, which exhibited significantly altered sensitivity only to normal Mg^2+^.

### NT and Mg^2+^ Sensitivity; Effects on Mg^2+^ Permeability and Allosteric Changes That Alter Mg^2+^ Binding

To help understand the results on Mg^2+^ modulation, we used homology modeling, estimated electrostatic potentials within the pore center, experimentally measured g_j_ and transjunctional Mg^2+^ fluxes and fit the electrophysiological and imaging data to mathematical models that took into account GJ channel gating and Mg^2+^ binding to open and/or closed states. Mg^2+^ flux data along with simultaneous measurements of g_j_, enabled assessment of total junctional membrane permeability normalized to the junctional conductance, thereby providing an estimate of open channel permeability to Mg^2+^.

Model fitting of the data showed that Model 5 with the Hill equation for Mg^2+^ binding provided the best fit according to values of AIC. The estimated Akaike weights (see Table 2) indicate that Model 5 with the Hill equation is almost twice as likely to be the best compared to the second best model, Model 4 with the Hill equation (see Table 2). In the latter model, hemichannel closure is caused by Mg^2+^ binding to the open state (the first step), which is followed by a Mg^2+^ independent transition to a second closed state (the second step). However, the estimated values of the parameters for Model 4 predict large differences between WT and some of the variants, such as E3Q (see Supplementary Material), which is not supported by the moderate differences observed experimentally (Figures 2–4). In addition, the Model 5 provided the lowest SSE for independent validation data on reductions in g_j_ in high symmetric Mg^2+^ concentrations, which was not included into the model fitting and analyses of AIC values. Thus, the combination of model fitting and independent data validation strongly indicates that Model 5 with Hill equation is the best candidate among the considered models.

Model fits indicate that the A13K variant is the only one that shows a substantive, ∼10-fold, reduction in Mg^2+^ permeability, which could contribute significantly to a reduced apparent affinity for Mg^2+^ binding. All the charge substitutions resulted in reduced negativity of the electrostatic profiles, and these changes correlated with the changes in Mg^2+^ permeability. However the correlation was only moderate, which indicates that changes in Mg^2+^ permeability are affected by other factors. For example, Figure 6D shows that A13K and H18K, which are located closer to the cytoplasm than E3Q and E8Q, exhibited a more robust decrease in Mg^2+^ permeability than would be predicted by a simple linear dependence on the reduced negativity.

Given that reduced Mg^2+^ permeability alone cannot explain the robust effects of NT substitutions on Mg^2+^ sensitivity, likely explanations include altered Mg^2+^ binding and/or altered stabilization of the Mg^2+^-bound closed state. As previously indicated, studies reported D47 in the E1 domain as an important component of a putative Mg^2+^ binding site (Palacios-Prado et al., 2014). In a GJ channel, this position in each hemichannel would be located deep in the pore, in the extracellular gap region, remote from NT. In order for NT substitutions to impact Mg^2+^ binding at D47, structural changes would need to translate from NT to E1. Early NMR studies of NT peptides composed of Cx32 sequence indicated that the NT domain has a helical component in its N-terminal end and forms on open turn in the vicinity of the G12 residue (Purnick et al., 2000a). This NT structure exhibited intrinsic flexibility that could serve to position the initial helical portion of the NT domain into the pore. Although this characterization was based on NMR studies of isolated NT peptides, the fundamental arrangement of the NT domain containing a turn was later confirmed by high resolution structures of Cx26 and Cx46/Cx50 GJ channels (Maeda et al., 2009; Myers et al., 2018) as depicted in the homology model for Cx36 in Figure 1. The turn that positions the N-terminal end of NT into the pore also explains the reported contributions of NT to voltage sensing (Verselis et al., 1994; Purnick et al., 2000b), which in GJs is mediated by residues positioned in the pore where they can sense the transjunctional field independent of the transmembrane field (Harris et al., 1981; Bukauskas and Verselis, 2004; Bargiello et al., 2018). Channel closure was associated with movement of the pore-forming region of NT toward the cytoplasm. The ultimate picture that has emerged is an NT structure that is dynamic and that participates in conformational changes that open and close GJ channels in response to transjunctional voltage and perhaps other stimuli. Although not yet understood in structural detail, a completed and equilibrated structure of a Cx26 hemichannel refined by molecular dynamics simulations suggested that gating of Cx channels involves rearrangement of a network of residues that interact electrostatically and through van der Waals forces to impact the positioning of a parahelical domain residing in a segment of E1. Based on a more recent cryo-EM structure of a Cx46/50 GJ channel resolved at a resolution of 1.9 Å (Flores et al., 2020), all-atom molecular dynamics simulations applied to a T39R cataract mutant in Cx50, which leads to increased hemichannel opening, suggested that stabilization of the open state of the hemichannel could occur through a direct interaction between NT and TM1, specifically through two alternative configurations, a D3-T39R salt bridge and a tripartite interaction involving G2, T39R and E42 (Tong et al., 2021). Thus, what is apparent is that NT and E1 domains are highly coupled, potentially through a combination of allosteric and direct interactions, that can plausibly link changes in NT with Mg^2+^ binding putatively assigned to reside in E1.

We note that a G46D charge substitution in Cx43, which aligns with D47 in Cx36, was able to confer a high Mg^2+^ sensitivity to Cx43 despite an NT domain that naturally contains a positively charged Lys at position 13. Thus, the effects of NT residues would appear to differ between Cx43 and Cx36, which is not surprising given that sequence differences between these Cxs likely result in differences in the patterns of residues comprising the electrostatic and van der Waals networks that govern Cx channel gating and likely contribute to the coupling of the NT and E1 domains. Conversely, the presence of charge alone at the D47 position in other Cxs does not necessarily confer Mg^2+^ sensitivity, again consistent with the integrative nature of the conformational changes that occur with Cx channel opening and closing. Moreover, the pore boundaries and overall electrostatic profiles of Cx channels are likely to differ, evident from the available structures for Cx26 and Cx46/Cx50 (Maeda et al., 2009; Myers et al., 2018), which can contribute to differences in Mg^2+^ permeability. In this view, Cx channels, although related in overall construction, likely differ in the positioning of residues within the network and the pore, explaining the wide heterogeneity in biophysical properties documented among various Cx channels and possible differential effects of individual amino acid substitutions in different Cx channels.

Although homology modeling using MODELLER and the available crystal structures of Cx26 and Cx46/50 provides a viable and established method for approximating the structure of Cx36, other algorithms are available to test alternative structural predictions for Cx36. For example, the protein folding algorithm AlphaFold (Senior et al., 2020) predicts a Cx36 NT domain that adopts a wide bend that does not pack well with TM1/E1 residues. This orientation is unlike the NT domains in the Cx26 and Cx46/50 crystal structures as well as in the NT domains of other Cx structures predicted by AlphaFold, in which the bend angle of the NT domain is steep allowing for interactions with TM1/E1 residues. Whether this difference is indicative of a unique conformational structure of Cx36 remains unknown. We note that AlphaFold shows that the predicted structure near the 13-14 positions in NT, which is the putative hinge region of the bend, shows low reliability (only ∼50 percent confidence level). Also, although chimeric studies in which the NT domain of Cx36 was substituted with Cx43 sequence produced functional channels, the reciprocal chimera, in which NT domain of Cx43 was substituted with Cx36 sequence failed to yield functional channels suggesting that there may be a sufficiently different NT structure for Cx36 such that it fails to be accommodated properly into a Cx43 background. In the event that the Cx36 NT structure adopts a unique orientation compared to other Cxs, the effects of the NT substitutions we examined here will be informative in providing mechanistic insights into NT structure and function.

Our modeling did not explicitly reveal changes in potential interactions between the residues at the substituted sites or at D47. However, our homology modeling was based on the Cx46/Cx50 structure, which is proposed to be in the open state, with the NT domain stabilized by anchoring hydrophobic interactions with TM1/TM2 helices (Myers et al., 2018; Yue et al., 2021). Thus, if Mg^2+^ binding occurs in the closed conformation, as suggested by our mathematical modeling data, we should not expect to detect any potential interactions in the homology model. Moreover, given the critical dependence on the structural template, it is likely that structural perturbations and alterations in side chain packing resulting from sequence divergence between Cx36 and Cx46/Cx50 would be lost in the approximated channel structure. Although Cxs can exhibit considerable sequence similarities, particularly in the more conserved NT, transmembrane and extracellular loop domains, phylogenetic analyses have divided Cxs into three main groups, with Cx36 belonging to the delta or Group IIIa designation (White et al., 2004; Cruciani and Mikalsen, 2007). Available structures Cx46/Cx50 and Cx26 represent Group I (Cx46/Cx50) and Group II (Cx26) connexins and, thus are more distant to Cx36. We chose to use the Cx46/Cx50 structure as a template due to a better estimated sequence similarity of more than 53 percent.

### Insights About Mg^2+^-Mediated Regulation of Cx36 Channels From Modeling Data

Electrophysiological studies of Cx36 GJ channels are hindered by their unusually low unitary conductance, which precludes direct measurement of open channel conductance and open probability. Our data showed that the same applies for all the Cx36 variants considered in this study. Thus, we combined macroscopic electrophysiological recordings with mathematical modeling to gain insights into channel behavior. Using different kinetic schemes of Mg^2+^-mediated channel gating, E8Q, A13K and H18K GJ channels were consistently predicted to be less permeable to Mg^2+^ compared to WT Cx36. The models also suggest that the observed differences in Mg^2+^-mediated changes in g_j_ can be adequately explained by a 3-state linear scheme. The best fit to the data was provided by the model in which the first transition represents Mg^2+^-independent gating between open and closed states in each of the hemichannels, and the second transition represents the binding of Mg^2+^ and stabilization of the closed hemichannel conformation. The estimated values of the transition rates associated with the Mg^2+^-independent transition (Table 2) suggest that it represents stochastic gating and can occur even in absence of V_j_ gradients. Such stochastic gating was demonstrated for Cx26 hemichannels for which single channel data is available (Sanchez et al., 2013). Modeling of data using our recently published gating model, 4SM, that takes into account contingent gating of two series hemichannels and the distribution of Vj across each hemichannel (Snipas et al., 2020) indicates that such stochastic gating at V_j_ = 0 mV would be sufficient to drive a Mg^2+^-dependent reduction in g_j_ caused by Mg^2+^ binding to the closed state. Moreover, as mentioned in the results, the estimated values of model parameters *k*_1_ and *k*_2_ of different variants correlated well with the observed changes in V_j_ sensitivity at high Mg^2+^ concentrations. This provides additional support for our model, in which Mg^2+^ ions stabilize hemichannel closure induced by the intrinsic V_j_-dependent gating. Interestingly, this scenario is very similar to the collapsed version of the mathematical model for the effects of Ca^2+^ on Cx46 hemichannels, in which Ca^2+^ was proposed to bind to and stabilize the closed conformation and in which Ca^2+^ binding is allosterically coupled to the voltage sensor (Pinto et al., 2017). Of note, the effects of Mg^2+^ on Cx46 hemichannels are similar to Ca^2+^, but with lower affinity.

Thus, Model 5 provides the best overall fit to the data on V_j_ gating and the observed Mg^2+^-induced changes in g_j_ among the considered candidate models. A simpler, two-state *O*↔*C* kinetic scheme together with the Hill equation to describe Mg^2+^ binding provided a good fit as well, but it could not reproduce the impeded recovery of g_j_ (data not shown). We acknowledge that whether Mg^2+^ binds to the closed and not to the open conformation of Cx36 GJ channels lacks direct experimental evidence. However, our model fitting together with data on Cx46 regulation by Ca^2+^ strengthens this possibility and may represent a common mechanism by which Cxs are regulated by divalent cations.

Given that we could not measure single channel events for Cx36 GJs, we cannot exclude effects of Mg^2+^ on unitary conductance. Intracellular Mg^2+^ was shown to affect unitary conductance of Cx50 GJ channels (Tejada et al., 2018). However, the changes over a 100-fold range of Mg^2+^ concentrations were relatively small and would not account for the effects observed for Cx36 GJs. In any case, we were unable to measure unitary events for A13K channels, which lack Mg^2+^ modulation, indicating the inherently small conductance characteristic of WT Cx36 was retained in A13K channels. Moreover, this issue was addressed in a study by Palacios et al. (Palacios-Prado et al., 2014), by taking advantage of a Cx43/Cx36 chimera with a large unitary conductance in which Mg^2+^ sensitivity was transferred by domain substitution. In that study, Mg^2+^ was shown to have no effect on unitary conductance, despite strong modulation of g_j_ indicating that Mg^2+^ acts by modulating channel gating. For these reasons, in this study, we only considered kinetic schemes in which hemichannel transits between fully open and closed states.

Mg^2+^-stabilization of the closed conformation requires access to the binding site when the channel is closed. All-atom molecular dynamics (MD) simulations of Cx26 hemichannels based on the Cx26 structure suggested that the reorganization of the electrostatic and van der Waals networks that is proposed to represent gating results in a substantial narrowing of the pore in the vicinity of the parahelical region in E1, which in Cx26 spans from E42 to F51 (Kwon et al., 2011; Bargiello et al., 2018). Assuming a conserved gating motif in all Cxs including Cx36, a constriction could occur near the putative Mg^2+^ binding site potentially allowing for such a state-dependent binding event. These MD simulations also suggested that the reorganization of the network could change the angle of the bend in the TM1/E1 region thereby changing the conformation at the cytoplasmic entrance, which could represent an aspect of the coupling between NT and E1 regions and thus the ability of NT and E1 mutations to affect Mg^2+^ sensitivity.

### The Existence of Hypothetical Deep-Closed State

Previous studies of Mg^2+^ modulation of Cx36 suggested that there may be a “deep-closed” conformation, in which both hemichannels of a Cx36 GJ channel are in a closed conformation resulting in the trapping of Mg^2+^ ions inside a pocket between two physical gates (Palacios-Prado et al., 2013). Such a scenario could leave Cx36 GJ channels in a stable and long-lived closed conformation. Our simulation data in Figure 5B showed that the probability of residing in a double-closed state (both hemichannels closed) is relatively high for Cx36 and most of the variants we studied, even in the absence of a trapping mechanism. However, when we simulated such a mechanism into our model by significantly reducing *k*_4_ transition rates when both hemichannels reached a Mg^2+^-bound closed state, the probability to reach a double-closed state exceeded 90% during 2-3 hours of simulation, even at physiological concentrations of Mg^2+^. Given that the half-life of a Cx36 GJ channel can be short and on the order of 3 hrs (Wang et al., 2015), such a hypothetical mechanism could provide an explanation, in part, for the generally low fraction of channels within a GJ plaque that appear to be functional at any given time, particularly those formed of Cx36 (Curti et al., 2012; Marandykina et al., 2013; Szoboszlay et al., 2016). Given that we did not observe a larger plaque size associated with A13K GJs (data not shown), which lack Mg^2+^-induced stabilization of the closed conformation, they may exhibit a higher fraction of functional channels, a possibility consistent with a significantly higher value for g_j,init_.

### NT Mutations

To our knowledge, there are no Cx36 mutations associated with disease that have been identified. However, mutations in 10 different human Cxs have been linked to over two dozen distinct genetic disorders with eight of them linked to mutations in Cx26 alone (Delmar et al., 2018; Srinivas et al., 2018). Of particular interest here, several Cx26 disease-causing missense mutations that result in syndromic forms of deafness have been shown to cluster in the NT and E1 domains. These mutant channels have been shown to function, either as hemichannels and/or as GJ channels, and exhibit a spectrum of aberrant functional properties (Lee and White, 2009; Sanchez and Verselis, 2014). Mutation at the G12 position in NT of Cx32 has been reported in association with Charcot-Marie-Tooth disease (Bergoffen et al., 1993) and in Cx30.3 with erythrokeratodermia variabilis (Richard et al., 1998). Mutations at several NT positions in Cx46 are associated with congenetical cataractogenesis (Berthoud and Ngezahayo, 2017). Given the structural configuration of the NT domain as the cytoplasmic vestibule of the pore, it is not surprising that NT mutations, through altered intra- and inter-subunit interactions, can alter the structure of NT and its inherent flexibility, thereby imparting longer-range effects in other parts of the channel. For Cx36, the interdependence between pH and Mg^2+^ modulation could be mediated by titration of NT residues that plausibly cause allosteric changes that affect the binding of Mg^2+^, putatively ascribed to the E1 domain, and/or by alterations in gating that affect the state-dependent nature of the binding of Mg^2+^ suggested by our modeling. Absent mutations, posttranslational modifications, such as acetylation of the N-terminal methionine that was suggested as mechanism for imparting differential permeability characteristics to Cx26 (Kwon et al., 2011), may also play a role in Cx36. Phosphorylation of Cx36 residues in cytoplasmic domains have been shown to occur in association with CaMKII and Ca^2+^ influx and to lead to increases the strength of electrical coupling (Del Corsso et al., 2012; Siu et al., 2021). Subsequent effects on Mg^2+^ sensitivity by phosphorylation by CaMKII or other post-translational modifications can also occur, thereby potentially linking Mg^2+^ modulation to neuronal activity and the metabolic status of the brain.

## Data Availability Statement

The raw data supporting the conclusions of this article will be made available by the authors, without undue reservation.

## Author Contributions

TK, LK, LG, MS, NP-P, and VV designed the research and analyzed the data. TK, LG, and LK performed the experiments. TK performed the homology modeling and molecular dynamic simulations. MS constructed and applied mathematical models. LG, LK, and NP-P critically revised the manuscript. TK, MS, and VV wrote the manuscript. All authors contributed to the article and approved the submitted version.

## Conflict of Interest

The authors declare that the research was conducted in the absence of any commercial or financial relationships that could be construed as a potential conflict of interest.

## Publisher’s Note

All claims expressed in this article are solely those of the authors and do not necessarily represent those of their affiliated organizations, or those of the publisher, the editors and the reviewers. Any product that may be evaluated in this article, or claim that may be made by its manufacturer, is not guaranteed or endorsed by the publisher.
