# Supplementary material for: The Amino Terminal Domain and Modulation of Connexin36 Gap Junction Channels by Intracellular Magnesium Ions
Source: Front Physiol. 2022 Feb 21;13:839223. doi: 10.3389/fphys.2022.839223 (PMC8899287; doi:10.3389/fphys.2022.839223)
Supplement: Supplementary file 1 [file Table_1.docx]

Supplementary Material

# Supplementary Data

**1.1 The description of mathematical models for Mg^2+^-mediated regulation of Cx36 channels**

**Model 1:** The kinetic scheme of the Model 1 is presented below:

$$O+{Mg}^{2+} \begin{matrix} k_{1} \\ \rightleftarrows\\ k_{2} \end{matrix} O­{Mg}^{2+}$$

$$O­{Mg}^{2+}+{Mg}^{2+} \begin{matrix} k_{3} \\ \rightleftarrows\\ k_{4} \end{matrix} C­{Mg}^{2+}­{Mg}^{2+}$$

Here $O$ denotes an open, Mg^2+^-free hemichannel, $O­{Mg}^{2+}$is Mg^2+^-bound open hemichannel, $C­{Mg}^{2+}­{Mg}^{2+}$is a closed hemichannel bound to two Mg^2+^ ions. Thus, the first transition of this model is Mg^2+^ binding to the open hemichannel, which results to a hemichannel-Mg^2+^ complex in an open conformation, while the second transition is another Mg^2+^-binding event to this Mg^2+^-bound hemichannel, which results to the closure of the .

The system of ordinary differential equations (ODE’s) describing Mg^2+^-binding and closure of a single hemichannel is presented below:

$$\left\{ \begin{aligned} \frac{d\left[ O \right]_{i}}{dt}=-k_{1}\cdot\left[ O \right]_{i}\cdot\left[ {Mg}^{2+} \right]_{i}+k_{2}{\cdot\left[ O­{Mg}^{2+} \right]}_{i} \\ \frac{d\left[ O­{Mg}^{2+} \right]_{i}}{dt}=k_{1}\cdot\left[ O \right]_{i}\cdot\left[ {Mg}^{2+} \right]_{i}-\left( k_{2}+k_{3}{\cdot\left[ {Mg}^{2+} \right]}_{i} \right){\cdot\left[ O­{Mg}^{2+} \right]}_{i}+k_{4}{\cdot\left[ C­{Mg}^{2+}­{Mg}^{2+} \right]}_{i} \\ \frac{d\left[ C­{Mg}^{2+}­{Mg}^{2+} \right]_{i}}{dt}=k_{3}{\cdot\left[ O­{Mg}^{2+} \right]}_{i}\cdot\left[ {Mg}^{2+} \right]_{i}-k_{4}{\cdot\left[ C­{Mg}^{2+}­{Mg}^{2+} \right]}_{i} \end{aligned} \right.$$

Here, *i* (*i* =1,2) denotes the hemichannel in each of two apposing cells. State variables $\left[ O \right]_{i}$, $\left[ O­{Mg}^{2+} \right]_{i}$ and $\left[ C­{Mg}^{2+}­{Mg}^{2+} \right]_{i}$ denote the probabilities that a hemichannel resides in an open Mg^2+^-free, an open Mg^2+^-bound and a closed state bound to two Mg^2+^ ions, respectively. These variables must be in the range [0;1] and satisfy the following conservation rule:

${\left[ O \right]_{i}+\left[ O­{Mg}^{2+} \right]}_{i}+\left[ C­{Mg}^{2+}­{Mg}^{2+} \right]_{i}=1$.

Based on the assumption that each apposing hemichannel can close the GJ channel pore, g_j_ can be expressed as $g_{j}=\left( \left[ O \right]_{1}+\left[ O­{Mg}^{2+} \right]_{1} \right)\cdot\left( \left[ O \right]_{2}+\left[ O­{Mg}^{2+} \right]_{2} \right)$.

The estimated parameters of this model for WT and mutated Cx36 channels are presented in Supplementary Table 1.

**Supplementary Table 1.** The estimated parameters of Model 1

| Cx | *k*_1_,  [min∙mM]^-1^ | *k*_2_,  min^-1^ | *k*_3_, [min∙mM]^-1^ | *k*_4_,  min^-1^ | *P,*  [min∙nS]^-1^ |
| --- | --- | --- | --- | --- | --- |
| WT | 386.96 | 155.13 | 0.0617 | 0.1737 | 0.0133 |
| E3Q | 393.71 | 92.05 | 0.0360 | 0.0460 | 0.0256 |
| E8Q | 402.21 | 0.0000 | 0.0219 | 0.0785 | 0.0076 |
| A13K | 403.17 | 0.0014 | 0.0112 | 0.7538 | 0.0011 |
| H18K | 399.89 | 0.0000 | 0.0338 | 0.2765 | 0.0074 |

**Model 1 with Hill equation:** Adding the Hill equation to the Model 1 results to the following kinetic scheme:

$$O+n_{1}\cdot{Mg}^{2+} \begin{matrix} k_{1} \\ \rightleftarrows\\ k_{2} \end{matrix} O­{Mg}^{2+}$$

$$O­{Mg}^{2+}+n_{2}\cdot{Mg}^{2+} \begin{matrix} k_{3} \\ \rightleftarrows\\ k_{4} \end{matrix} C­{Mg}^{2+}­{Mg}^{2+}$$

Here *n*_1_ and *n*_2_ denotes the Hill coefficients for Mg^2+^ binding events.

The system of ODE’s would then be modified as follows:

$$\left\{ \begin{aligned} \frac{d\left[ O \right]_{i}}{dt}=-k_{1}\cdot\left[ O \right]_{i}\cdot\left[ {Mg}^{2+} \right]_{i}^{n_{1}}+k_{2}{\cdot\left[ O­{Mg}^{2+} \right]}_{i} \\ \frac{d\left[ O­{Mg}^{2+} \right]_{i}}{dt}=k_{1}\cdot\left[ O \right]_{i}\cdot\left[ {Mg}^{2+} \right]_{i}^{n_{1}}-\left( {k_{2}+k}_{3}\cdot\left[ {Mg}^{2+} \right]_{i}^{n_{2}} \right){\cdot\left[ O­{Mg}^{2+} \right]}_{i}+k_{4}{\cdot\left[ C­{Mg}^{2+}­{Mg}^{2+} \right]}_{i} \\ \frac{d\left[ C­{Mg}^{2+}­{Mg}^{2+} \right]_{i}}{dt}=k_{3}{\cdot\left[ O­{Mg}^{2+} \right]}_{i}\cdot\left[ {Mg}^{2+} \right]_{i}^{n_{2}}-k_{4}{\cdot\left[ C­{Mg}^{2+}­{Mg}^{2+} \right]}_{i} \end{aligned} \right.$$

The estimated parameters of the Model 1 with Hill equation are presented in Supplementary Table 2.

**Supplementary Table 2.** The estimated parameters of Model 1 with Hill equation

| Cx | *k*_1_,  [min∙mM]^-1^ | *k*_2_,  min^-1^ | *k*_3_, [min∙mM]^-1^ | *k*_4_,  min^-1^ | *n*_1_ | *n*_2_ | *P,*  [min∙nS]^-1^ |
| --- | --- | --- | --- | --- | --- | --- | --- |
| WT | 386.96 | 155.11 | 0.0611 | 0.1739 | 1.00 | 1.01 | 0.0133 |
| E3Q | 393.71 | 92.05 | 0.0362 | 0.0459 | 1.00 | 1.00 | 0.0256 |
| E8Q | 402.23 | 0.0000 | 0.0222 | 0.0749 | 1.00 | 0.99 | 0.0076 |
| A13K | 403.03 | 0.1563 | 0.0110 | 0.9491 | 1.00 | 1.11 | 0.0011 |
| H18K | 3999.83 | 0.0000 | 0.0397 | 0.1203 | 0.00 | 0.75 | 0.0074 |

**Model 2:** The kinetic scheme of the Model 2 is presented below:

$$O+{Mg}^{2+} \begin{matrix} k_{1} \\ \rightleftarrows\\ k_{2} \end{matrix} O­{Mg}^{2+}$$

$$O­{Mg}^{2+} \begin{matrix} k_{3} \\ \rightleftarrows\\ k_{4} \end{matrix} C­{Mg}^{2+}$$

Here $O$ denotes an open, Mg^2+^-free hemichannel, $O­{Mg}^{2+}$ is a Mg^2+^-bound open hemichannel, and $C­{Mg}^{2+}$is a closed Mg^2+^-bound hemichannel. Thus, the first transition of this model is Mg^2+^ binding to the open hemichannel, which results to a hemichannel-Mg^2+^ complex in an open conformation, while the second transition is a Mg^2+^-independent transition, which results in closure of the hemichannel.

The system of ordinary differential equations (ODE’s) describing Mg^2+^-binding and closure of a single hemichannel is presented below:

$$\left\{ \begin{aligned} \frac{d\left[ O \right]_{i}}{dt}=-k_{1}\cdot\left[ O \right]_{i}\cdot\left[ {Mg}^{2+} \right]_{i}+k_{2}{\cdot\left[ O­{Mg}^{2+} \right]}_{i} \\ \frac{d\left[ O­{Mg}^{2+} \right]_{i}}{dt}=k_{1}\cdot\left[ O \right]_{i}\cdot\left[ {Mg}^{2+} \right]_{i}-\left( k_{2}+k_{3} \right){\cdot\left[ O­{Mg}^{2+} \right]}_{i}+k_{4}{\cdot\left[ C­{Mg}^{2+} \right]}_{i} \\ \frac{d\left[ C­{Mg}^{2+} \right]_{i}}{dt}=k_{3}{\cdot\left[ O­{Mg}^{2+} \right]}_{i}-k_{4}{\cdot\left[ C­{Mg}^{2+}­{Mg}^{2+} \right]}_{i} \end{aligned} \right.$$

Here, *i* (*i* =1,2) denotes the hemichannel in each of two apposing cells. State variables $\left[ O \right]_{i}$, $\left[ O­{Mg}^{2+} \right]_{i}$ and $\left[ C­{Mg}^{2+} \right]_{i}$ denote the probabilities that a hemichannel resides in an open Mg^2+^-free, an open Mg^2+^-bound and a closed Mg^2+^-bound state, respectively. These variables must be in the range [0;1] and satisfy the following conservation rule:

${\left[ O \right]_{i}+\left[ O­{Mg}^{2+} \right]}_{i}+\left[ C­{Mg}^{2+} \right]_{i}=1$.

Based on the assumption that each apposing hemichannel can close a GJ channel pore, g_j_ can be expressed as $g_{j}=\left( \left[ O \right]_{1}+\left[ O­{Mg}^{2+} \right]_{1} \right)\cdot\left( \left[ O \right]_{2}+\left[ O­{Mg}^{2+} \right]_{2} \right)$.

The estimated parameters of this model for WT and mutated Cx36 channels are presented in Table S3.

**Supplementary Table 3.** The estimated parameters of Model 2

| Cx | *k*_1_,  [min∙mM]^-1^ | *k*_2_,  min^-1^ | *k*_3_,  min^-1^ | *k*_4_,  min^-1^ | *P,*  [min∙nS]^-1^ |
| --- | --- | --- | --- | --- | --- |
| WT | 2.01 | 3847.78 | 120.37 | 0.2034 | 0.0134 |
| E3Q | 1.00 | 3560.91 | 130.20 | 0.0567 | 0.0257 |
| E8Q | 14.83 | 3849.45 | 5.94 | 0.0835 | 0.0076 |
| A13K | 0.66 | 2211.06 | 46.81 | 0.9773 | 0.0011 |
| H18K | 379.72 | 194.30 | 0.25 | 0.4229 | 0.0072 |

**Model 2 with Hill equation:** Adding the Hill equation to the Model 2 results to the following kinetic scheme:

$$O+n\cdot{Mg}^{2+} \begin{matrix} k_{1} \\ \rightleftarrows\\ k_{2} \end{matrix} O­{Mg}^{2+}$$

$$O­{Mg}^{2+} \begin{matrix} k_{3} \\ \rightleftarrows\\ k_{4} \end{matrix} C­{Mg}^{2+}$$

Here *n* denotes the Hill coefficient for Mg^2+^ binding event.

The system of ODE’s would then be modified as follows:

$$\left\{ \begin{aligned} \frac{d\left[ O \right]_{i}}{dt}=-k_{1}\cdot\left[ O \right]_{i}\cdot\left[ {Mg}^{2+} \right]_{i}^{n}+k_{2}{\cdot\left[ O­{Mg}^{2+} \right]}_{i} \\ \frac{d\left[ O­{Mg}^{2+} \right]_{i}}{dt}=k_{1}\cdot\left[ O \right]_{i}\cdot\left[ {Mg}^{2+} \right]_{i}^{n}-\left( k_{2}+k_{3} \right){\cdot\left[ O­{Mg}^{2+} \right]}_{i}+k_{4}{\cdot\left[ C­{Mg}^{2+} \right]}_{i} \\ \frac{d\left[ C­{Mg}^{2+} \right]_{i}}{dt}=k_{3}{\cdot\left[ O­{Mg}^{2+} \right]}_{i}-k_{4}{\cdot\left[ C­{Mg}^{2+} \right]}_{i} \end{aligned} \right.$$

The estimated parameters of the Model 2 with the Hill equation are presented in Supplementary Table 4.

**Supplementary Table 4.**The estimated parameters of Model 2 with Hill equation

| Cx | *k*_1_,  [min∙mM]^-1^ | *k*_2_,  min^-1^ | *k*_3_,  min^-1^ | *k*_4_,  min^-1^ | *n* | *P,*  [min∙nS]^-1^ |
| --- | --- | --- | --- | --- | --- | --- |
| WT | 1.63 | 3832.27 | 122.77 | 0.2019 | 1.10 | 0.0134 |
| E3Q | 0.50 | 3729.89 | 258.13 | 0.0590 | 1.05 | 0.0258 |
| E8Q | 1.46 | 3903.25 | 63.13 | 0.0850 | 0.98 | 0.0076 |
| A13K | 0.07 | 3902.32 | 97.31 | 0.0959 | 1.44 | 0.0011 |
| H18K | 1.22 | 3661.75 | 338.17 | 0.1914 | 0.00 | 0.0070 |

**Model 3:** The kinetic scheme of the Model 3 is presented below:

$$O+{Mg}^{2+} \begin{matrix} k_{1} \\ \rightleftarrows\\ k_{2} \end{matrix} C­{Mg}^{2+}$$

$$C­{Mg}^{2+}+{Mg}^{2+} \begin{matrix} k_{3} \\ \rightleftarrows\\ k_{4} \end{matrix} C­{Mg}^{2+}­{Mg}^{2+}$$

Here $O$ denotes an open, Mg^2+^-free hemichannel, $C­{Mg}^{2+}$ is a closed hemichannel bound to a single Mg^2+^ ion, and $C­{Mg}^{2+}­{Mg}^{2+}$ is a closed hemichannel bound to two Mg^2+^ ions. Thus, the first transition of this model is Mg^2+^ binding to an open hemichannel, which results in a hemichannel-Mg^2+^ complex in a closed conformation, while the second transition is another Mg^2+^ binding event, which could be interpreted as providing better stabilization of the closed conformation of the hemichannel.

The system of ordinary differential equations (ODE’s) describing Mg^2+^-binding and closure of a single hemichannel is presented below:

$$\left\{ \begin{aligned} \frac{d\left[ O \right]_{i}}{dt}=-k_{1}\cdot\left[ O \right]_{i}\cdot\left[ {Mg}^{2+} \right]_{i}+k_{2}{\cdot\left[ C­{Mg}^{2+} \right]}_{i} \\ \frac{d\left[ C­{Mg}^{2+} \right]_{i}}{dt}=k_{1}\cdot\left[ O \right]_{i}\cdot\left[ {Mg}^{2+} \right]_{i}-\left( k_{2}+k_{3}{\cdot\left[ {Mg}^{2+} \right]}_{i} \right){\cdot\left[ C­{Mg}^{2+} \right]}_{i}+k_{4}{\cdot\left[ C­{Mg}^{2+}­{Mg}^{2+} \right]}_{i} \\ \frac{d\left[ C­{Mg}^{2+}­{Mg}^{2+} \right]_{i}}{dt}=k_{3}{\cdot\left[ C­{Mg}^{2+} \right]}_{i}\cdot\left[ {Mg}^{2+} \right]_{i}-k_{4}{\cdot\left[ C­{Mg}^{2+}­{Mg}^{2+} \right]}_{i} \end{aligned} \right.$$

Here, *i* (*i* =1,2) denotes the hemichannel in each of two apposing cells. State variables $\left[ O \right]_{i}$, $\left[ C­{Mg}^{2+} \right]_{i}$ and $\left[ C­{Mg}^{2+}­{Mg}^{2+} \right]_{i}$ denote the probabilities that a hemichannel resides in an open Mg^2+^-free, or a closed state, bound to a single and two Mg^2+^ ions, respectively. These variables must be in the range [0;1] and satisfy the following conservation rule:

${\left[ O \right]_{i}+\left[ C­{Mg}^{2+} \right]}_{i}+\left[ C­{Mg}^{2+}­{Mg}^{2+} \right]_{i}=1$.

Based on the assumption that each apposing hemichannel can close the GJ channel pore, g_j_ can be expressed as $g_{j}=\left[ O \right]_{1}\cdot\left[ O \right]_{2}$.

The estimated parameters of this model for WT and mutated Cx36 channels are presented in Supplementary Table 5.

**Supplementary Table 5.** The estimated parameters of Model 3

| Cx | *k*_1_,  [min∙mM]^-1^ | *k*_2_,  min^-1^ | *k*_3_, [min∙mM]^-1^ | *k*_4_,  min^-1^ | *P,*  [min∙nS]^-1^ |
| --- | --- | --- | --- | --- | --- |
| WT | 0.0654 | 0.2846 | 0.0142 | 0.0000 | 0.0135 |
| E3Q | 0.0364 | 0.0878 | 0.0211 | 0.0000 | 0.0257 |
| E8Q | 0.0238 | 0.1698 | 0.0302 | 0.0000 | 0.0076 |
| A13K | 0.0293 | 3.9745 | 0.0502 | 0.2353 | 0.0011 |
| H18K | 0.0323 | 0.2439 | 0.0599 | 4004.17 | 0.0074 |

**Model 3 with Hill equation:** Adding the Hill equation to the Model 3 results to the following kinetic scheme:

$$O+n_{1}\cdot{Mg}^{2+} \begin{matrix} k_{1} \\ \rightleftarrows\\ k_{2} \end{matrix} C­{Mg}^{2+}$$

$$C­{Mg}^{2+}+n_{2}\cdot{Mg}^{2+} \begin{matrix} k_{3} \\ \rightleftarrows\\ k_{4} \end{matrix} C­{Mg}^{2+}­{Mg}^{2+}$$

Here *n*_1_ and *n*_2_ denotes the Hill coefficients for Mg^2+^ binding events.

The system of ODE’s would then be modified as follows:

$$\left\{ \begin{aligned} \frac{d\left[ O \right]_{i}}{dt}=-k_{1}\cdot\left[ O \right]_{i}\cdot\left[ {Mg}^{2+} \right]_{i}^{n_{1}}+k_{2}{\cdot\left[ C­{Mg}^{2+} \right]}_{i} \\ \frac{d\left[ C­{Mg}^{2+} \right]_{i}}{dt}=k_{1}\cdot\left[ O \right]_{i}\cdot\left[ {Mg}^{2+} \right]_{i}^{n_{1}}-\left( {k_{2}+k}_{3}\cdot\left[ {Mg}^{2+} \right]_{i}^{n_{2}} \right){\cdot\left[ C­{Mg}^{2+} \right]}_{i}+k_{4}{\cdot\left[ C­{Mg}^{2+}­{Mg}^{2+} \right]}_{i} \\ \frac{d\left[ C­{Mg}^{2+}­{Mg}^{2+} \right]_{i}}{dt}=k_{3}{\cdot\left[ C­{Mg}^{2+} \right]}_{i}\cdot\left[ {Mg}^{2+} \right]_{i}^{n_{2}}-k_{4}{\cdot\left[ C­{Mg}^{2+}­{Mg}^{2+} \right]}_{i} \end{aligned} \right.$$

The estimated parameters of the Model 3 with the Hill equation are presented in Supplementary Table 6.

**Supplementary Table 6**. The estimated parameters of Model 3 with Hill equation

| Cx | *k*_1_,  [min∙mM]^-1^ | *k*_2_,  min^-1^ | *k*_3_, [min∙mM]^-1^ | *k*_4_,  min^-1^ | *n*_1_ | *n*_2_ | *P,*  [min∙nS]^-1^ |
| --- | --- | --- | --- | --- | --- | --- | --- |
| WT | 0.0654 | 0.6176 | 0.4878 | 0.1250 | 1.08 | 0.00 | 0.0135 |
| E3Q | 0.0364 | 0.2656 | 0.6666 | 0.0000 | 1.05 | 0.00 | 0.0257 |
| E8Q | 0.0259 | 0.1720 | 0.0302 | 0.0000 | 0.95 | 1.09 | 0.0076 |
| A13K | 0.0293 | 4.2323 | 0.0385 | 0.2134 | 1.03 | 1.09 | 0.0011 |
| H18K | 0.0323 | 0.2439 | 271.68 | 999.13 | 0.94 | 0.00 | 0.0074 |

**Model 4:** The kinetic scheme of the Model 4 is presented below:

$$O+{Mg}^{2+} \begin{matrix} k_{1} \\ \rightleftarrows\\ k_{2} \end{matrix} C_{1}­{Mg}^{2+}$$

$$C_{1}­{Mg}^{2+} \begin{matrix} k_{3} \\ \rightleftarrows\\ k_{4} \end{matrix} C_{2}­{Mg}^{2+}$$

Here $O$ denotes an open, Mg^2+^-free hemichannel, $C_{1}­{Mg}^{2+}$ is a Mg^2+^-bound hemichannel in the first closed state, and $C_{2}­{Mg}^{2+}$ is a Mg^2+^-bound hemichannel residing in the second, more stably closed state (i.e., a deep-closed state). Thus, the first transition of this model is Mg^2+^ binding to the open hemichannel, which results to a hemichannel-Mg^2+^ complex in a closed conformation, while the second transition is a gating event resulting the hemichannel in the deep-closed conformation.

The system of ordinary differential equations (ODE’s) describing Mg^2+^-binding and closure of a single hemichannel is presented below:

$$\left\{ \begin{aligned} \frac{d\left[ O \right]_{i}}{dt}=-k_{1}\cdot\left[ O \right]_{i}\cdot\left[ {Mg}^{2+} \right]_{i}+k_{2}{\cdot\left[ C_{1}­{Mg}^{2+} \right]}_{i} \\ \frac{d\left[ C_{1}­{Mg}^{2+} \right]_{i}}{dt}=k_{1}\cdot\left[ O \right]_{i}\cdot\left[ {Mg}^{2+} \right]_{i}-\left( k_{2}+k_{3} \right){\cdot\left[ C_{1}­{Mg}^{2+} \right]}_{i}+k_{4}{\cdot\left[ C_{2}­{Mg}^{2+} \right]}_{i} \\ \frac{d\left[ C_{2}­{Mg}^{2+} \right]_{i}}{dt}=k_{3}{\cdot\left[ C_{1}­{Mg}^{2+} \right]}_{i}-k_{4}{\cdot\left[ C_{2}­{Mg}^{2+} \right]}_{i} \end{aligned} \right.$$

Here, *i* (*i* =1,2) denotes the hemichannel in each of two apposing cells. State variables $\left[ O \right]_{i}$, $\left[ C_{1}­{Mg}^{2+} \right]_{i}$ and $\left[ C_{2}­{Mg}^{2+} \right]_{i}$ denote the probabilities that a hemichannel resides in an open Mg^2+^-free or two closed Mg^2+^-bound states, respectively. These variables must be in the range [0;1] and satisfy the following conservation rule:

${\left[ O \right]_{i}+\left[ C_{1}­{Mg}^{2+} \right]}_{i}+\left[ C_{2}­{Mg}^{2+} \right]_{i}=1$.

Based on the assumption that each apposing hemichannel can close the GJ channel pore, g_j_ can be expressed as $g_{j}=\left[ O \right]_{1}\cdot\left[ O \right]_{2}$.

The estimated parameters of this model for WT and mutated Cx36 channels are presented in Supplementary Table 7.

**Supplementary Table 7**. The estimated parameters of Model 4

| Cx | *k*_1_,  [min∙mM]^-1^ | *k*_2_,  min^-1^ | *k*_3_, [min∙mM]^-1^ | *k*_4_,  min^-1^ | *P,*  [min∙nS]^-1^ |
| --- | --- | --- | --- | --- | --- |
| WT | 0.0796 | 0.7406 | 0.6360 | 0.1742 | 0.0134 |
| E3Q | 0.1445 | 33.50 | 10.17 | 0.0527 | 0.0258 |
| E8Q | 0.1001 | 14.96 | 3.50 | 0.0431 | 0.0076 |
| A13K | 0.2780 | 64.38 | 1.21 | 0.4317 | 0.0011 |
| H18K | 66.58 | 3334.23 | 0.8667 | 0.0515 | 0.0075 |

**Model 4 with Hill equation:** Adding the Hill equation to the Model 3 results to the following kinetic scheme:

$$O+n\cdot{Mg}^{2+} \begin{matrix} k_{1} \\ \rightleftarrows\\ k_{2} \end{matrix} C_{1}­{Mg}^{2+}$$

$$C_{1}­{Mg}^{2+} \begin{matrix} k_{3} \\ \rightleftarrows\\ k_{4} \end{matrix} C_{2}­{Mg}^{2+}$$

Here *n* and denotes the Hill coefficient for Mg^2+^ binding events.

The system of ODE’s would then be modified as follows:

$$\left\{ \begin{aligned} \frac{d\left[ O \right]_{i}}{dt}=-k_{1}\cdot\left[ O \right]_{i}\cdot\left[ {Mg}^{2+} \right]_{i}^{n}+k_{2}{\cdot\left[ C_{1}­{Mg}^{2+} \right]}_{i} \\ \frac{d\left[ C_{1}­{Mg}^{2+} \right]_{i}}{dt}=k_{1}\cdot\left[ O \right]_{i}\cdot\left[ {Mg}^{2+} \right]_{i}^{n_{1}}-\left( {k_{2}+k}_{3} \right){\cdot\left[ C_{1}­{Mg}^{2+} \right]}_{i}+k_{4}{\cdot\left[ C_{2}­{Mg}^{2+} \right]}_{i} \\ \frac{d\left[ C_{2}­{Mg}^{2+} \right]_{i}}{dt}=k_{3}{\cdot\left[ C_{1}­{Mg}^{2+} \right]}_{i}-k_{4}{\cdot\left[ C_{2}­{Mg}^{2+} \right]}_{i} \end{aligned} \right.$$

The estimated parameters of the Model 4 with the Hill equation are presented in Supplementary Table 8.

**Supplementary Table 8**. The estimated parameters of Model 4 with Hill equation

| Cx | *k*_1_,  [min∙mM]^-1^ | *k*_2_,  min^-1^ | *k*_3_, [min∙mM]^-1^ | *k*_4_,  min^-1^ | *n* | *P,*  [min∙nS]^-1^ |
| --- | --- | --- | --- | --- | --- | --- |
| WT | 0.0666 | 0.7559 | 0.6717 | 0.1840 | 1.09 | 0.0135 |
| E3Q | 0.0666 | 9.99 | 7.77 | 0.1059 | 1.09 | 0.0262 |
| E8Q | 0.0715 | 7.27 | 2.58 | 0.0344 | 0.99 | 0.0076 |
| A13K | 0.0079 | 2.53 | 0.6013 | 0.5281 | 1.44 | 0.0011 |
| H18K | 0.1446 | 0.9493 | 2.02 | 1.1482 | 0.10 | 0.0071 |

**Model 5:** The kinetic scheme of the Model 5 is presented below:

$$O \begin{matrix} k_{1} \\ \rightleftarrows\\ k_{2} \end{matrix} C$$

$$C+{Mg}^{2+} \begin{matrix} k_{3} \\ \rightleftarrows\\ k_{4} \end{matrix} C­{Mg}^{2+}$$

Here $O$ denotes an open, Mg^2+^-free hemichannel, $C$ is a Mg^2+^-free hemichannel in a closed conformation, and $C­{Mg}^{2+}$ is a Mg^2+^-bound hemichannel residing in a closed state. Thus, the first transition of this model is a Mg^2+^-independent gating event, while the second transition is a stabilization of the closed state caused by Mg^2+^ binding to the closed conformation.

The system of ODEs, describing Mg^2+^-binding and closure of a single hemichannel is presented below:

$$\left\{ \begin{aligned} \frac{d\left[ O \right]_{i}}{dt}=-k_{1}\cdot\left[ O \right]_{i}+k_{2}{\cdot\left[ C \right]}_{i} \\ \frac{d\left[ C \right]_{i}}{dt}=k_{1}{\cdot\left[ O \right]}_{i}-\left( k_{2}+k_{3}\cdot\left[ {Mg}^{2+} \right]_{i} \right){\cdot\left[ C \right]}_{i}+k_{4}{\cdot\left[ C­{Mg}^{2+} \right]}_{i} \\ \frac{d\left[ C­{Mg}^{2+} \right]_{i}}{dt}=k_{3}\cdot\left[ C \right]_{i}\cdot\left[ {Mg}^{2+} \right]_{i}-k_{4}{\cdot\left[ C­{Mg}^{2+} \right]}_{i} \end{aligned} \right.$$

Here, *i* (*i* =1,2) denotes the hemichannel in each of two apposing cells, cell-1 or cell-2 and *n* is the Hill coefficient for the binding of Mg^2+^ ions. State variables $\left[ O \right]_{i}$, $\left[ C \right]_{i}$ and $\left[ C­{Mg}^{2+} \right]_{i}$ denote the probabilities that a hemichannel resides in an open, closed or Mg^2+^-stabilized closed state, respectively. These variables must be in the range [0;1] and satisfy the following conservation rule:

${\left[ O \right]_{i}+\left[ C \right]}_{i}+\left[ C­{Mg}^{2+} \right]_{i}=1$.

Based on the assumption that each apposing hemichannel can close the GJ channel pore, g_j_ can be expressed as $g_{j}=\left[ O \right]_{1}\cdot\left[ O \right]_{2}$. The estimated parameters of this model for WT and mutated Cx36 channels are presented in Table 3.

Based on the assumption that each apposing hemichannel can close the GJ channel pore, g_j_ can be expressed as $g_{j}=\left[ O \right]_{1}\cdot\left[ O \right]_{2}$.

The estimated parameters of this model for WT and mutated Cx36 channels are presented in Supplementary Table 9.

**Supplementary Table 9**. The estimated parameters of Model 5

| Cx | *k*_1_,  min^-1^ | *k*_2_,  min^-1^ | *k*_3_, [min∙mM]^-1^ | *k*_4_,  min^-1^ | *P,*  [min∙nS]^-1^ |
| --- | --- | --- | --- | --- | --- |
| WT | 2.94 | 347.05 | 6.89 | 0.1874 | 0.0134 |
| E3Q | 1.36 | 397.57 | 10.12 | 0.0500 | 0.0257 |
| E8Q | 0.69 | 383.56 | 1.02 | 0.0308 | 0.0076 |
| A13K | 0.70 | 398.04 | 12.89 | 1.69 | 0.0011 |
| H18K | 0.14 | 0.53 | 0.07 | 0.0328 | 0.0071 |

**Model 5 with Hill equation**

Adding the Hill equation to the Model 3 results to the following kinetic scheme:

$$O \begin{matrix} k_{1} \\ \rightleftarrows\\ k_{2} \end{matrix} C$$

$$C+n{\cdot Mg}^{2+} \begin{matrix} k_{3} \\ \rightleftarrows\\ k_{4} \end{matrix} C­{Mg}^{2+}$$

Here *n* and denotes the Hill coefficient for Mg^2+^ binding event.

The system of ODE’s would then be modified as follows:

$$\left\{ \begin{aligned} \frac{d\left[ O \right]_{i}}{dt}=-k_{1}\cdot\left[ O \right]_{i}+k_{2}{\cdot\left[ C \right]}_{i} \\ \frac{d\left[ C \right]_{i}}{dt}=k_{1}{\cdot\left[ O \right]}_{i}-\left( k_{2}+k_{3}\cdot\left[ {Mg}^{2+} \right]_{i}^{n} \right){\cdot\left[ C \right]}_{i}+k_{4}{\cdot\left[ C­{Mg}^{2+} \right]}_{i} \\ \frac{d\left[ C­{Mg}^{2+} \right]_{i}}{dt}=k_{3}\cdot\left[ C \right]_{i}\cdot\left[ {Mg}^{2+} \right]_{i}^{n}-k_{4}{\cdot\left[ C­{Mg}^{2+} \right]}_{i} \end{aligned} \right.$$

The estimated parameters of the Model 5 with the Hill equation are presented in Supplementary Table 10.

**Supplementary Table 10**. The estimated parameters of Model 5 with Hill equation

| Cx | *k*_1_,  [min]^-1^ | *k*_2_,  min^-1^ | *k*_3_, [min∙mM]^-1^ | *k*_4_,  min^-1^ | *n* | *P,*  [min∙nS]^-1^ |
| --- | --- | --- | --- | --- | --- | --- |
| WT | 5.35 | 386.57 | 3.2770 | 0.1802 | 1.12 | 0.0135 |
| E3Q | 3.52 | 392.28 | 3.2366 | 0.0417 | 1.08 | 0.0258 |
| E8Q | 5.88 | 271.07 | 0.7168 | 0.0197 | 1.07 | 0.0076 |
| A13K | 3.05 | 179.34 | 0.0026 | 0.0637 | 2.84 | 0.0011 |
| H18K | 9.80 | 337.60 | 3.2391 | 0.2008 | 0.11 | 0.0072 |

The values of ΔAIC for model fitting and SSE for independent validation data are presented in Supplementary Table 11.

**Supplementary Table 11**. The fit (ΔAIC) and independent validation measures (SSE) of different models applied to electrophysiological and fluorescent imaging data for different Cx36 variants

|  | **Model 1** | | **Model 2** | | **Model 3** | | **Model 4** | | **Model 5** | |
| --- | --- | --- | --- | --- | --- | --- | --- | --- | --- | --- |
|  | ΔAIC | SSE | ΔAIC | SSE | ΔAIC | SSE | ΔAIC | SSE | ΔAIC | SSE |
| WT | -212.93 | 0.0055 | -200.35 | 0.0092 | -205.22 | 0.0131 | -211.61 | 0.0186 | -201.41 | 0.0091 |
| E3Q | -187.43 | 0.0184 | -184.16 | 0.0155 | -185.15 | 0.0153 | -185.85 | 0.0145 | -184.08 | 0.0162 |
| E8Q | -206.41 | 0.0031 | -207.11 | 0.0027 | -210.63 | 0.0071 | -213.33 | 0.0047 | -221.27 | 0.0060 |
| A13K | -216.90 | 0.0248 | -214.57 | 0.0265 | -235.26 | 0.0126 | -219.23 | 0.0271 | -211.45 | 0.0331 |
| H18K | -77.88 | 0.0361 | -166.34 | 0.0797 | -78.26 | 0.0388 | -82.41 | 0.0567 | -175.75 | 0.0025 |
| **Σ** | -901.55 | 0.0879 | -972.53 | 0.1336 | -914.52 | 0.0869 | -912.43 | 0.1216 | -993.96 | 0.0669 |
|  | **Model 1, Hill** | | **Model 2, Hill** | | **Model 3, Hill** | | **Model 4, Hill** | | **Model 5, Hill** | |
|  | ΔAIC | SSE | ΔAIC | SSE | ΔAIC | SSE | ΔAIC | SSE | ΔAIC | SSE |
| WT | -208.95 | 0.0054 | -203.15 | 0.007 | -212.84 | 0.0156 | -215.07 | 0.0158 | -205.60 | 0.0081 |
| E3Q | -183.43 | 0.0183 | -183.82 | 0.0185 | -185.72 | 0.0166 | -182.55 | 0.0174 | -183.73 | 0.0226 |
| E8Q | -202.68 | 0.0028 | -203.76 | 0.0028 | -211.43 | 0.0056 | -211.79 | 0.0049 | -221.97 | 0.0104 |
| A13K | -216.50 | 0.0224 | -192.45 | 0.0121 | -231.81 | 0.0111 | -230.14 | 0.0099 | -223.74 | 0.0044 |
| H18K | -90.95 | 0.0528 | -158.14 | 0.0148 | -78.21 | 0.0349 | -170.41 | 0.0024 | -176.21 | 0.0015 |
| **Σ** | -902.51 | 0.1017 | -941.33 | 0.0552 | -920.01 | 0.0838 | -1009.95 | 0.0504 | -1011.25 | 0.0470 |

# Supplementary Figures





**Supplementary Figure 1.** The comparison of Cx36 wild-type, A13K and H18K channels sensitivity to transjunctional voltage (V_j_). (**A**) V_j_ ramp protocol used in electrophysiological recordings. (B) The averaged changes in junctional conductance (g_j_) presented as g_j_-V_j_ curves for wild-type Cx36 and its variants (n = 6).
